# Supplementary material for: Steps Towards Alcohol Misuse Prevention Programme (STAMPP): a school-based and community-based cluster randomised controlled trial
Source: BMJ Open. 2018 Mar 9;8(3):e019722. doi: 10.1136/bmjopen-2017-019722 (PMC5879538; doi:10.1136/bmjopen-2017-019722)
Supplement: Supplementary file 1 [file bmjopen-2017-019722supp001.pdf]

ONLINE SUPPLEMENTARY MATERIAL

INTERVENTION CONTENT

The Classroom component of the intervention was composed of six lessons in the second year of High school (Phase one), and four lessons in the third year (Phase two). The content of these is detailed below.

Phase 1

Lesson 1: Alcohol True or False (10 statements); Introduction to what is meant by 'Units' of alcohol; Introduction to the extent of harm that alcohol misuse can cause.

Lesson 2: Making Choices – why people choose to drink (and assessing the merit of those choices); Making Choices – why people may choose not to drink; Introduction to Alcohol and the Body.

Lesson 3: Units of Alcohol – more detail including unit content of drinks; Relating consumption to consequences; Short Quiz to recap information.

Lesson 4: Blood Alcohol Concentration; Alcohol harms in various societal contexts (with other drugs, in families, in communities, driving, and sexual behaviour).

Lesson 5: Exercise – 'What would you do to reduce harms?' Critical examination of alcohol and the Media.

Lesson 6: Real Life Scenarios, plus recap.

Phase 2

Lesson 1: Brief recap from previous year; Alcohol and the Body – long term versus short term; Quiz.

Lesson 2: A night out – examining dangers, laws, problems, pressures and consequences.

Lesson 3: Vulnerability – two scenarios examined from the point of view of 'victim', friends, and 'perpetrator'; Planning for a safe night out with friends.

Lesson 4: Ranking Risk; What would you advise a friend to do?

STAMPP – FULL PRIMARY OUTCOME MODELS, SECONDARY OUTCOMES AND SUBGROUP ANALYSES

FULL PRIMARY OUTCOME MODELS

For reasons of space, the full primary outcome models were not presented in the main text. Table S1 presents the parameter estimates from a two level random intercepts logistic regression model for the heavy episodic drinking (HED) primary outcome at T3.

Table S1. Primary outcome (HED) outcome analysis at + 33 months

	Estimate	S.E.	OR	P value
**ITT Complete case analysis**				
Within level				
Baseline HED	1.395	0.093	4.036	<0.001
Between Level				
Intervention Arm	-0.516	0.102		<0.001
Free School Meals (tertile)	0.239	0.073		0.001
School Type				
Boys School Dummy	-0.186	0.200		0.35
Girls School Dummy	-0.546	0.266		0.04
Location (NI)	0.422	0.109		<0.001
School level residual variance	0.176	0.035		<0.001
Threshold (BngT3$1)	1.574	0.124		<0.001

Table S2 gives the parameter estimates from a two level random intercepts negative binomial model for the drinking harms primary outcome at T3.

Table S2. Primary outcome (ARH) outcome analysis at + 33 months

	Estimate	S.E.	P value
**Complete case analysis**			
Within level			
Baseline Harms	0.211	0.011	<0.001
Between Level			
Intervention Arm	-0.101	0.083	0.222
Free School Meals (tertile)	0.168	0.061	0.006
School Type			
Boys School Dummy	-0.083	0.204	0.685
Girls School Dummy	-0.380	0.236	0.107
Location	0.433	0.082	<0.001
Residual variances	0.115	0.026	<0.001
Intercept (HarmsT3)	-0.042	0.093	0.649
Dispersion (HarmsT3)	3.563	0.207	<0.001

SECONDARY OUTCOMES

A range of secondary outcomes were also examined within the study. These included the primary outcomes assessed at T2:

Heavy episodic drinking (HED) (T2): Self-reported alcohol use defined as self-reported consumption of >5 drinks, assessed at +24 months (T2) from baseline. This was dichotomised at none/one or more occasions. This outcome was assessed via a two level random intercepts logistic regression model. Around 12.4% of respondents reported HED at T2 using this measure. In the intervention arm HED was reported by 10.9% (N=573) and in the control arm by 13.9% (N=722).

Alcohol related harms (T2): The number of self-reported harms (harms caused by own drinking) assessed at +24 months (T2) from baseline. Items included harms such as getting into a physical fight or being sick after drinking. The outcome was a count of the number of discrete harms reported (0-16) and was assessed by a two level random intercepts negative binomial model. In the intervention arm 74.3% reported no drinking harms, while in the control arm 71.5% reported no harms.

In addition, a number of secondary outcomes at T3 and T2 were also examined, including:

Lifetime drinking (T3): Whether the pupils had ever consumed a full drink of alcohol at +33 months (T3) (two level random intercepts logistic regression model).

Last year drinking (T3): Whether the pupils had consumed a full drink of alcohol in the last year, assessed at +33 months (T3) (two level random intercepts logistic regression model).

Last month Drinking (T3): Whether the pupils had consumed a full drink of alcohol in the last month, assessed at +33 months (T3) (two level random intercepts logistic regression model).

Harm from others (T3 and T2): The number of self-reported harms experienced that were the result of other people's drinking, assessed at both +33 months (T3) and +24 months (T2) from baseline (two level random intercepts negative binomial models). Harms included being hit or having property damaged by someone who had been drinking.

Age of onset (T3 and T2): Self-reported age at which respondent first consumed a full drink, assessed at both +33 months (T3) and +24 months (T2) from baseline (two level random intercepts Cox regression model).

Unsupervised drinking (T3 and T2): Whether the pupils were permitted, by their parents(s), to consume alcohol (with small group of friends or at parties) with no adult present, assessed at both +33 months (T3) and +24 months (T2) from baseline (two level random intercepts logistic regression model).

Number of drinks consumed (T3 and T2): Pupils were asked whether they usually drank from a range of different alcohol drinks (beer, alcopops, spirits cider, wine, *Buckfast* [a popular brand of fortified wine, with caffeine], others) and if so, how much did they usually drink. The values for each drink were summed together to give a total. As the underlying items continued decimals the total value was multiplied by 10 to create whole numbers.

The secondary outcome analysis also included covariates at level 1 (individual) and level 2 (school) where appropriate:

The models use for the secondary outcome were similar to those employed in the primary outcome analysis with a single level one covariate, and the treatment indicator and stratification variables used in the randomisation as level two covariates.

Level 1 covariate

Relevant baseline drinking variable (T0): For each outcome, the corresponding baseline observations were included in the model. Mean imputation was used to impute values for those respondents who were missing on this variable. The only model not to include a baseline covariate was age of onset.

Level 2 covariates

Treatment Arm: This was a binary covariate in which schools in the control arm were coded 0 and schools in the intervention arm were coded 1.

Free school meals (Randomisation stratification factor): Schools were classified into three groups based on free school meal provision. The allocation was based on a tertile split based on information provided by head teachers on the proportion of pupils in receipt of free school meals: *Low* Free School Meal Provision (0-15.4%), *Moderate* Free School Meal Provision (15.5-30.4%), *High* Free School Meal Provision (30.5% and above).

School type (Randomisation stratification factor): Given the larger number of schools in Northern Ireland, an additional stratification factor was used in the randomisation. This was school type (all boys' school/ all girls' school/coeducation school). Schools in

Glasgow/Inverclyde were all assigned to the co-education type. This indicator was used represented by two dummy variables (co-education was the comparison category).

Location: A dummy variable was generated to indicate the location of the schools (Northern Ireland/Scotland).

Results from the analysis of secondary outcomes

Table S3 presents the random intercept models for the primary outcomes at +24 months. The baseline measures were significant, as was location. For the HED outcomes both free school meals (tertile split) and school type were significant. The intervention arm was significant at a 0.05 level (β=-0.241; p=0.041). However, it failed to reach the much stricter threshold used in the primary analysis (0.025). It should be noted that the HED indicator used at +33 months, and as specified in the DAP, was different that that used at +24 months. In particular, this measure did not use gender specific splits, referred to drinks rather than units, and did not provide any visual guides to help with the estimation of amount consumed. This suggests that the significant intervention effect may have been partly dependent on the precision of the measurement instrument used to collect the primary outcome data. The age at which differences in HED were assessed may have been important when assessing intervention outcomes.

Table S3. Secondary analysis: primary outcomes at +24 months

	Estimate	S.E.	OR	P value
**HED T2 (ITT CC population, logistic model)**				
Within level				
Baseline HED	1.891	0.101	6.623	<0.001
Between Level				
Treatment Arm	-0.241	0.118		0.041
Free School Meals (tertile)	0.308	0.079		<0.001

School Type				
Boys School Dummy	-0.708	0.297		0.02
Girls School Dummy	-0.608	0.186		0.001
Location	0.732	0.134		<0.001
Residual variance	0.214	0.047		<0.001
Threshold (BngT2$1)	2.698	0.144		<0.001
**Harms to Self T2 (ITT CC population, negative binomial model)**				
Within level				
Baseline Harms drinking	0.297	0.016		<0.001
Between Level				
Treatment Arm	-0.144	0.118		0.22
Free School Meals (tertile)	0.162	0.086		0.06
School Type				
Boys School Dummy	-0.247	0.302		0.42
Girls School Dummy	-0.246	0.200		0.22
Location	0.716	0.132		<0.001
Residual variance	0.267	0.054		<0.001
Intercepts (SHarmsT2)	-0.779	0.133		<0.001
Dispersion	4.478	0.304		<0.001

Table S4 presents the outcome models for the additional secondary outcomes assessed at T3. The treatment indicator was not significant in any of these models.

Table S4. Secondary outcomes at +33 months

	Estimate	S.E.	OR	P value
**Lifetime drinking T3 (ITT CC population, logistic model)**				
Within level				
Baseline HED	2·070	0·081	7·922	<0·001
Between Level				
Treatment Arm	-0·125	0·102		0·22
Free School Meals (tertile)	0·040	0·070		0·57
School Type				
Boys School Dummy	-0·182	0·209		0·384
Girls School Dummy	-0·501	0·233		0·031
Location	0·597	0·113		<0·001
Residual variance	0·209	0·035		<0·001
Threshold (LifeT3$1)	0·419	0·114		<0·001

Table S4. Secondary outcomes at +33 months (cont.)

	Estimate	S.E.	OR	P value
**Last year drinking T3 (ITT CC population, logistic model)**				
Within level				
Baseline Last year drinking	1·822	0·086	6·187	<0·001
Between Level				
Treatment Arm	-0·126	0·096		0·19
Free School Meals (tertile)	0·011	0·065		0·87

School Type				
Boys School Dummy	-0·176	0·211		0·40
Girls School Dummy	-0·401	0·229		0·08
Location	0·615	0·105		<0·001
Residual variances	0·177	0·032		<0·001
Threshold (LYearT3$1)	0·485	0·103		<0·001
**Last month drinking T3 (ITT CC population, logistic model)**				
Within level				
Baseline Last month drinking	1·329	0·114	3·779	<0·001
Between Level				
Treatment Arm	-0·149	0·094		0·11
Free School Meals (tertile)	0·114	0·069		0·10
School Type				
Boys School Dummy	-0·333	0·213		0·12
Girls School Dummy	-0·330	0·237		0·16
Location	0·381	0·104		<0·001
Residual variances	0·148	0·028		<0·001
Threshold (LMonthT3$1)	1·459	0·102		<0·001
**Harms from others drinking T3 (ITT CC population, Neg Bin model)**				
Within level				
Baseline Harms (others)	0·330	0·016		<0·001
Between Level				
Treatment Arm	0·000	0·057		0·10
Free School Meals (tertile)	0·077	0·042		0·07
School Type				
Boys School Dummy	0·117	0·116		0·31
Girls School Dummy	-0·070	0·172		0·68
Location	0·167	0·063		0·01
Residual variance	0·050	0·014		<0·001
Dispersion	1·301	0·071		<0·001
Intercept	-0·733	0·061		<0·001
**Age of onset T3 (ITT CC population, Cox regression model)**				
Between Level				
Treatment Arm	-0·095	0·067		0·16
Free School Meals (tertile)	0·054	0·047		0·25
School Type				
Boys School Dummy	-0·299	0·146		0·04
Girls School Dummy	-0·407	0·145		0·01
Location	0·344	0·075		<0·001
Residual variance	0·097	0·017		< 0·001

Table S4. Secondary outcomes at +33 months (cont.)

	Estimate	S.E.	OR	P value
**Unsupervised drinking T3 (ITT CC population Logistic model)**				
Within level				
Baseline unsupervised drinking	1.782	0.091	5.940	<0.001
Between Level				
Treatment Arm	-0.142	0.092		0.123
Free School Meals (tertile)	0.128	0.067		0.058

School Type				
Boys School Dummy	0.002	0.207		0.992
Girls School Dummy	-0.236	0.236		0.318
Location	0.564	0.102		<0.001
Residual variance	0.148	0.029		<0.001
Threshold (Unsuper$1)	0.148	0.029		<0.001
**Number of drinks T3 (ITT CC population NB model)**				
Within level				
Baseline number of drinks	0.126	0.009		<0.001
Between Level				
Treatment Arm	-0.078	0.075		0.297
Free School Meals (tertile)	0.123	0.048		0.011
School Type				
Boys School Dummy	-0.277	0.181		0.127
Girls School Dummy	-0.167	0.177		0.346
Location	0.363	0.075		<0.001
Residual variances	0.073	0.020		<0.001
Intercept (NumDrkT3)	3.521	0.082		<0.001
Dispersion (NumDrkT3)	5.371	0.306		<0.001

Note: The logistic regression multilevel models were estimated using a logit link function and the MLR estimator. The Cox regression model uses a non-parametric baseline hazard function and a profile likelihood estimation method

Table S5 presents the models for the secondary outcomes assessed at T2. Again, the treatment indicator was not significant in any of these models.

Table S5. Secondary outcomes at +24 months

	Estimate	S.E.	OR	P value
**Harms from others drinking T2 (ITT CC population, Neg Bin model)**				
Within level				
Baseline Harms (others)	0·421	0.017		<0.001
Between Level				
Treatment Arm	-0·058	0.060		0.33
Free School Meals (tertile)	0·132	0.044		0.003
School Type				
Boys School Dummy	0·144	0.108		0.18
Girls School Dummy	0·075	0.119		0.53
Location	0·255	0.071		<0.001
Residual variance	0·058	0.011		<0.001
Dispersion	1·032	0.078		<0.001
Intercept	-1·079	0.069		<0.001

Table S5. Secondary outcomes at +24 months

**Age of onset T2 (ITT CC population, Cox regression model)**			
Between Level			
Treatment Arm	-0·055	0.074	0.46
Free School Meals (tertile)	0·084	0.048	0.08
School Type			
Boys School Dummy	-0·528	0.197	0.007

Girls School Dummy	-0·453	0.169		0.007
Location	0·408	0.083		<0.001
Residual variance	0·176	0.028		<0.01
**Unsupervised drinking T2 (ITT CC population, Logistic model)**				
Within level				
Baseline unsupervised drinking	2·114	0.097	8.285	<0.001
Between Level				
Treatment Arm	-0·087	0.100		0.39
Free School Meals (tertile)	0·166	0.066		0.01
School Type				
Boys School Dummy	-0·306	0.217		0.16
Girls School Dummy	-0·207	0.135		0.12
Location	0·669	0.112		<0.001
Residual variance	0·170	0.038		<0.001
Threshold (Unsuper$1)	1·883	0.118		<0.001
**Number of drinks T2 (ITT CC population, NB model)**				
Within level				
Baseline unsupervised	0·170	0.013		<0.001
Between Level				
Treatment Arm	-0·088	0.096		0.36
Free School Meals (tertile)	0·125	0.068		0.07
School Type				
Boys School Dummy	-0·574	0.259		0.03
Girls School Dummy	-0·181	0.147		0.22
Location	0·583	0.105		<0.001
Residual variances	0·153	0.035		<0.001
Intercept (NumDrkT2)	2·836	0.106		<0.001
Dispersion (NumDrkT2)	5·671	0.340		<0.001

Note: The logistic regression multilevel models were estimated using a logit link function and the MLR estimator. The Cox regression model uses a non-parametric baseline hazard function and a profile likelihood estimation method

Subgroup analyses

To explore differential treatment effects on the primary and secondary outcome measures, pre-specified interaction terms were fitted between trial arm and baseline measures thought to predict the effect of treatment. These were:

- Age, in months, of pupil at baseline;
- Gender;
- Socioeconomic status (using the proportion of free school meals indicator);
- Alcohol use behaviour at baseline – ever use, last year use, age of onset, and context of use (abstainer/supervised/unsupervised);
- and in NI, a Grammar/Secondary school analysis.

Both the relevant covariate and interaction term were included in the model as a level 1 (within level) covariates. In all the subgroup analysis models estimated the corresponding interaction terms were all non-significant.
