# Supplementary material for: Steps Towards Alcohol Misuse Prevention Programme (STAMPP): a school-based and community-based cluster randomised controlled trial
Source: BMJ Open. 2018 Mar 9;8(3):e019722. doi: 10.1136/bmjopen-2017-019722 (PMC5879538; doi:10.1136/bmjopen-2017-019722)
Supplement: Author's manuscript [file bmjopen-2017-019722.draft_revisions.pdf]

BMJ Open

BMJ Open is committed to open peer review. As part of this commitment we make the peer review history of every article we publish publicly available.

When an article is published we post the peer reviewers' comments and the authors' responses online. We also post the versions of the paper that were used during peer review. These are the versions that the peer review comments apply to.

The versions of the paper that follow are the versions that were submitted during the peer review process. They are not the versions of record or the final published versions. They should not be cited or distributed as the published version of this manuscript.

BMJ Open is an open access journal and the full, final, typeset and author-corrected version of record of the manuscript is available on our site with no access controls, subscription charges or pay-per-view fees (http://bmjopen.bmj.com).

If you have any questions on BMJ Open's open peer review process please email info.bmjopen@bmj.com

BMJ Open

Steps towards alcohol misuse prevention programme (STAMPP): a school and community based cluster randomised controlled trial

Journal:	*BMJ Open*
Manuscript ID	bmjopen-2017-019722
Article Type:	Research
Date Submitted by the Author:	22-Sep-2017

Complete List of Authors:	McKay, Michael; Psychological Sciences<br>Agus, Ashley; Northern Ireland Clinical Trials Unit<br>Cole, Jonathan; University of Liverpool, Psychological Sciences<br>Doherty, Paul; Northern Ireland Clinical Trials Unit<br>Foxcroft, David; Oxford Brookes University UK<br>Harvey, Séamus; School of Sport, Health, and Exercise Sciences<br>Murphy, Lynn; Northern Ireland Clinical Trials Unit<br>Percy, Andrew; Queens University , School of Social Sciences, Education, and Social Work<br>Sumnall, Harry; Liverpool John Moores University, Public Health Institute

Keywords:	alcohol, school based intervention, prevention, alcohol related harm, universal prevention, adolescents

SCHOLARONE™
Manuscripts

For peer review only

Steps towards alcohol misuse prevention programme (STAMPP): a school and community based cluster randomised controlled trial

Corresponding author: Michael McKay[1 #3];

Email: Michael.McKay@liverpool.ac.uk

Tel: 0044 7875778186

Authors:

Michael McKay [1, #3] (teejaymck@hotmail.com), Ashley Agus [2] (ashley.agus@nictu.hscni.net); Jon Cole [3] (j.c.cole@liv.ac.uk); Paul Doherty [2] (paul.doherty@nictu.hscni.net); David Foxcroft [4] (david.foxcroft@brookes.ac.uk); Séamus Harvey [1,#6] (harveyseamus@gmail.com); Lynn Murphy [2] (lynn.murphy@nictu.hscni.net); Andrew Percy [5] (a.percy@qub.ac.uk); Harry Sumnall [1] (h.sumnall@ljmu.ac.uk).

[1] Public Health Institute, Liverpool John Moores University, UK

[2] Northern Ireland Clinical Trials Unit, The Royal Hospitals, Belfast, UK

[3] Department of Psychological Sciences, University of Liverpool, UK

[4] Psychology, Social Work and Public Health, Oxford Brookes University, UK

[5] School of Social Sciences, Education, and Social Work, Queen's University Belfast, UK

[6] School of Sport, Health and Exercise Sciences, University of Bangor, UK

# Current address

For peer review only - http://bmjopen.bmj.com/site/about/guidelines.xhtml

Abstract (Word count: 300)

Objectives: To assess the effectiveness of a combined classroom curriculum and parental intervention (The Steps Towards Alcohol Misuse Prevention Programme; STAMPP), compared to alcohol education as normal (EAN), in reducing self-reported heavy episodic drinking (HED) and alcohol-related harms (ARH) in school children.

Setting: 105 High schools in Northern Ireland (NI) and in Scotland.

Participants: Schools were stratified by free school meal provision. Schools in NI were also stratified by school type (male/female/co-educational). Eligible students were in school year 8/S1 (aged 11-12) at baseline in June 2012.

Intervention: A classroom-based alcohol education intervention, coupled with a brief alcohol intervention for parents/carers.

Primary Outcomes: The study had two primary outcomes at +33 months; the prevalence of self-reported HED in the previous 30 days and the number of self-reported ARHs in the previous six months.

Results: At 33 months data were available for 5,160 intervention and 5,073 control students (HED outcome), and 5,234 and 5,146 students (ARH outcome) respectively. Of the full sample (those who completed a questionnaire at either baseline or 12 months, N=12,738), 10,405 also completed the questionnaire at 33 months (81.7%). Fewer students in the STAMPP group reported HED compared to EAN (17% versus 26%; odds ratio=0·60, 95% CI 0·49-0·73). There was no difference in the number of

For peer review only - http://bmjopen.bmj.com/site/about/guidelines.xhtml

self-reported ARHs (incident rate ratio = 0·92, CI 0·78-1.05). Although the classroom component was largely delivered as intended, there was low uptake of the parental component. There were no reported adverse effects.

Conclusions: Results suggest that STAMPP could be an effective school-based program to reduce the prevalence of HED in young people. Whilst we did not find a reduction in ARH, it is plausible that effects on harms would manifest later.

Trial Registration: The trial was registered, number ISRCTN47028486 (http://www.isrctn.com/ISRCTN47028486). The date of trial registration was 23/09/2011, and school recruitment began 01/11/2011.

Article Summary

Strengths and Limitations.

- All data are longitudinal;
- The sample size was very large and attrition relatively low;
- Participants were independently randomised;
- Some of those involved in fieldwork were not blind to participant condition;
- Overall levels of alcohol-related harm were low.

Keywords: alcohol; prevention; school based intervention; alcohol related harm; universal prevention; adolescents

For peer review only - http://bmjopen.bmj.com/site/about/guidelines.xhtml

Introduction

Adolescence is a period when young people experiment with alcohol, and as they age the amount and frequency of consumption increases.(1) Research has shown that family socialisation factors such as approval of adolescent drinking and the provision of alcohol in the home predicts drinking among adolescents and young adults (2-4) An earlier onset of self-reported drunkenness and the establishment of regular alcohol drinking is associated with a greater risk of adult alcohol-related problems.(5) There are also clear geographic and socioeconomic differences in the burden alcohol places on the population, and these are closely associated with other major indicators of ill health and health inequalities. (6-8)

Previous literature reviews have highlighted a lack of high quality trials of universal school-based universal alcohol prevention programmes, and few approaches studied have shown positive intervention effects.(9-15) However, while reviews have been unable to recommend any single prevention initiative, many have concluded that interventions that develop social skills appear to be superior to those that seek to enhance only knowledge.(10-13) Guidance issued by the National Institute for Health and Care Excellence (NICE) in the UK in 2007 called for partnerships between schools and other stakeholders in efforts to prevent misuse.(16) Reviews of universal alcohol prevention in family settings suggest that activities supporting parenting skills, including establishing clear boundaries or rules and parental monitoring, may be effective.(9, 17, 18) Primary studies also suggest that when combined with a school-based alcohol curriculum, provision of advice to parents about setting strict rules around alcohol consumption reduces adolescent drinking.(19, 20)

For peer review only - http://bmjopen.bmj.com/site/about/guidelines.xhtml

The Steps Towards Alcohol Misuse Prevention Programme (STAMPP) intervention combined a culturally adapted intervention based on the School Health and Alcohol Harm Reduction Project (SHARHP)(21) curriculum with a researcher-developed brief parental intervention based on the Swedish Örebro Prevention Program.(22) SHAHRP is an example of a resistance skills training programme, and includes elements of alcohol-specific personal and social skills training. (23-26) In accordance with the theoretical assumptions underlying such programmes, it includes three main strategies : (i) teaching students to recognise high-risk situations, (ii) increasing the awareness of external influences on behaviour, and (iii) combining self-control (i.e. the ability to control responses, to interrupt undesired behavioural tendencies and refrain from acting upon them) with refusal skills training (i.e. in order to improve self-efficacy in avoiding unhealthy behaviours, but not with the consequence of social disadvantage for the young person with their peers). The knowledge delivered through SHAHRP (e.g. lessons on effects of alcohol, description of alcohol units) was not assumed to have direct preventative effects, but instead hypothesised to shape and alcohol attitudes and support situation-specific decision making. The parental component was based on research indicating that restrictive parenting practices (e.g., monitoring of children's alcohol use, healthy attitudes towards alcohol, alcohol rule-setting) was associated with reduced prevalence of children's alcohol use (20). When this approach was delivered alongside a classroom intervention in the Dutch PAS, programme effect was mediated through children's perceptions of parental rules, child self-efficacy, and child self-control. (27)

It was hypothesised that fewer students in schools delivering STAMPP would self-report: (i) past 30-day heavy episodic drinking (HED) at final follow-up (33

For peer review only

For peer review only - http://bmjopen.bmj.com/site/about/guidelines.xhtml

months from baseline); and (ii) fewer self-reported alcohol-related harms (ARH) at final follow-up than those in schools delivering alcohol education as normal (EAN). These primary aims of the research trial were to assess whether STAMPP was effective in reducing self-reporting of these two indicators of alcohol use.

Materials and Methods

Study design

This was a cluster randomised controlled trial (cRCT) of school children in Northern Ireland (NI) and Glasgow/Inverclyde Education Authority (Scotland) areas in the United Kingdom (UK) with schools as the unit of randomisation. The research was approved by Liverpool John Moores University Research Ethics Committee (11/HEA/097). The trial protocol is available from http://www.nets.nihr.ac.uk/projects/phr/10300209.

Participants

The sampling frame comprised all mainstream post primary schools in NI (excluding those within the Eastern Health Board due to existing delivery of SHAHRP in that area) and in Glasgow/Inverclyde Local Authorities. All schools in the sampling frame were assessed for satisfaction of the inclusion criteria and willingness to participate in the trial.

A total of 105 schools were invited to participate in the trial, and all accepted; 70 in NI, 30 in Glasgow Local Authority and five in Inverclyde Local Authority. Inclusion criteria were schools in NI and Scotland that taught students in school year 8/S1 in the academic year 2011/2012 (aged 11/12 at randomisation). Exclusion

For peer review only - http://bmjopen.bmj.com/site/about/guidelines.xhtml

criteria were schools that did not include students in the specified school year, or only provided non-mainstream or vocational education (e.g. pupil referral units, further education colleges). Individual students with special educational needs in mainstream classrooms were excluded at the discretion of teachers as the intervention materials had not been developed for use with this population.

Participants were eligible students in the randomised schools, who consented to participate. Opt in consent was obtained from school head-teachers/principals before randomisation. Opt out consent from participants and their parents/guardians was obtained after randomisation. No schools withdrew from the trial and no pupils or parents/carers withdrew consent. Data was collected under examination-like conditions on school premises.

Randomisation and blinding

Schools were randomly assigned (1:1) to receive STAMPP or alcohol EAN before baseline data were collected. Randomisation was performed by an independent statistician blind to the identity of the schools. All schools were stratified on Free School Meal Provision (FSM; low/moderate/high), which was taken as a proxy for socio-economic status. Schools in NI were also stratified by school-type (male/female/co-educational).

Schools, students, intervention trainers and delivery staff (teachers) were not blind to study condition. Data collection was undertaken by a team of researchers that included the trial manager and research assistants, some of whom were not blind to study condition.

For peer review only - http://bmjopen.bmj.com/site/about/guidelines.xhtml

Data analysis of primary and secondary outcomes was undertaken by the trial statistician who was blinded to the study condition.

Procedures

STAMPP combined a school-based skills development curriculum, and a brief parental intervention designed to support parents in setting family rules around drinking (see Table 1 for overview of the intervention). The classroom component of STAMPP was based on the SHAHRP intervention and culturally adapted for the settings of delivery.(28) It combined skills training, education, and activities designed to encourage positive behavioural change.(21) It was a curriculum-based programme delivered in two phases over a two year period. As part of the trial, the first phase was delivered when students were in school year 9/S2 (age 12-13 years) and the second phase was delivered during the subsequent year.

The parental component of STAMPP was developed by the trial team and was based on the programme structure of Koutakis and colleagues (22), and Koning and colleagues. (19, 20) The component differed in two main ways to these earlier programmes. Firstly, as part of STAMPP, delivery of a single parental component coincided with the delivery of phase two of the classroom curriculum, whereas in Koutakis and Koning, parents' evenings were held several times over the intervention delivery phase. Secondly, the session was partly based upon guidelines included in the UK Chief Medical Officers' 2009 guidelines for drinking in childhood (29). All intervention pupil parents, regardless of whether they had attended the evening or not,

For peer review only - http://bmjopen.bmj.com/site/about/guidelines.xhtml

were mailed an information leaflet a few weeks after the parental session which reinforced the discussion points.

For peer review only

For peer review only - http://bmjopen.bmj.com/site/about/guidelines.xhtml

200 **Table 1.** Stages in the STAMPP Trial

201

Stage	Description
Recruitment of schools	• Schools in Glasgow Local Authority (n = 30) were recruited as a complete group following negotiations with Education Services<br>• Schools in Inverclyde (n = 5) were recruited following a meeting with the Headteachers/Principals to discuss the practicalities of the trial.<br>• Schools in Northern Ireland (n = 70) were recruited individually in the following process: letter of information; follow-up telephone call; individual meeting with Headteacher/ Principals; agree yes/no.
Training of teachers	• One-day training events were held in each study site before both phases of delivery of the classroom component. Training for the following academic year (from September onwards) took place in the preceding June.<br>• Training involved lectures on alcohol (e.g. effects of alcohol use; prevalence rates; risk and protective factors for alcohol use), sharing experiences on previous delivery of the programme, and skills rehearsal for each of the SHAHRP lessons.<br>• Training involved examination of each of the SHAHRP Lessons which covered: Myths about Alcohol; Units of Alcohol; Reasons why people do/don't drink; Alcohol and the Body; Consequences of 'levels' of drinking; Blood Alcohol Concentration; Social and Personal Harms; Alcohol Policy; Alcohol and the Media; Advice for Teenagers; A 'Night Out'; Pressures faced by Young Drinkers; Scenario-based discussion.<br>• Each lesson was scheduled to last one lesson period (approximately 40 minutes) and delivered once a week

For peer review only - http://bmjopen.bmj.com/site/about/guidelines.xhtml

	• Teachers were provided with support materials (CD-ROMS, workbooks) at each training session to help implement the lessons.
Intervention Period	• The intervention period was September to November in both academic years. Phase One involved six lessons and Phase Two, four lessons. Schools were asked to complete all lessons within the three-month delivery window in both phases.<br>• The Parental Brief Intervention coincided with delivery of Phase Two when the children were in their third year of secondary school and took place in the evening on Intervention school premises The intervention included a brief presentation on the UK Chief Medical Officers' guidelines on alcohol use by young people, and a discussion on setting family rules on alcohol. All Intervention student parents, regardless of whether they had attended the evening or not, were mailed a leaflet which reinforced these points a few weeks after the parental session<br>• Final data collection for the primary outcome took place one year after all elements of the intervention had been delivered.

202

For peer review only - http://bmjopen.bmj.com/site/about/guidelines.xhtml

The control group participants continued with alcohol EAN within their school, which included standard personal, social, and health education, but would not be uniform across all such schools. Parents/carers of control students did not receive the STAMPP intervention or materials, but may have been exposed to alcohol intervention activities in the community as part of independent provision.

Questionnaires were administered to participants at baseline in June 2012 and at three follow-ups: 12, 24, and 33 months. All students that were present at baseline or joined participating schools prior to delivery of Phase 1 of the intervention were included in the analyses. Parents/carers were asked to complete a short postal questionnaire, which coincided with delivery of the information leaflet. Alcohol rules were assessed using a 10-item scale to measuring the degree to which parents/carers permitted their children to consume alcohol in various situations, such as 'in the absence of parents at home' or 'at a friend's party' ($\alpha = 0.86$-0.90). (30) Parental alcohol self-efficacy was assessed using a three item scale assessing the level of confidence the parent/carer had in their own ability to prevent their child from drinking ($\alpha = 0.67$).(31) This data was collected to inform future mediation analysis and is not reported here.

Outcomes

The study had two primary outcomes at 33 months; (i) the prevalence of self-reported HED drinking in the previous 30 days (HED defined as the consumption of ≥6 units [males]/ ≥4.5 units [females] on one or more occasions) and (ii) the number of self-reported harms (caused by own drinking) in the previous six months in students. Pre-specified secondary outcomes are described in the online supplementary material,

For peer review only - http://bmjopen.bmj.com/site/about/guidelines.xhtml

except for those related to the cost-effectiveness analysis which will be reported elsewhere. The original primary outcome was self-reported frequency of consumption of >5 'drinks' in a single drinking episode. However, concerns arose because it became clear that '>5 drinks' could refer to drinks of different alcohol strength and volume. As the objective of the intervention was to reduce HED, the primary outcome was changed to consumption of ≥6 units for males, and ≥4.5 units for females – both are 1.5 times the Chief Medical Officer's maximum daily guideline for adults,(29) and this was ratified by the independent Study Steering Committee. This change was implemented before the final wave of data collection, before unblinding, and before any analysis of trial outcome measures at any data collection point had been undertaken.

To assess the HED primary outcome, participants were presented with pictorial prompts of how much alcohol ≥6/≥4.5 UK units represents. Pictures presented the most popular drinks consumed in the two study areas and respondents were asked to report the frequency of consuming this amount of alcohol over the previous month. Harms associated with own use of alcohol were measured using a 16-item scale developed for the Australian SHAHRP trial (internal consistency 0.9). (32) Participants were asked to indicate on a Likert scale how many times in the past six months they had experienced the individual harm. For example, participants were asked to report frequency of having a hangover after drinking, or if they had got into a physical fight when drinking.

Statistical analysis

For peer review only - http://bmjopen.bmj.com/site/about/guidelines.xhtml

It was calculated that a sample size of 90 schools (45 per study arm; 80 students per school) would be powerful enough (80%; α= 0.05; ICC = 0.09 based on data from the Belfast Youth Development Study (33)) to detect a standardised effect size of $\delta = 0.2$, or a 10% absolute reduction in risk (51% vs. 41%) for the primary outcome of HED. Assuming 20% attrition within each cluster (from 100 to 80 students), the target sample size was 90 schools and 9000 students at baseline.

Summary statistics on school and student recruitment, withdrawal and dropout were collated for both trial arms and reported as a participant flow diagram for reporting of cRCT (Fig 1). Outcome measure scores from the questionnaires were summarised and tabulated for the trial arms.

The outcome analysis was an Intention to Treat (ITT) analysis using the Complete Case (CC) population such that all cases were assessed regardless of intervention and intervention dosage. Logistic regression models estimated the association between STAMPP and the odds of self-reported HED. Negative binomial regression models estimated the association between STAMPP and the number of AHR. All models included school-level random intercepts to account for correlation due to clustering of students within schools. All models adjusted for factors used to stratify randomization and the outcome's corresponding value at baseline. For details of analysis of secondary outcomes please see the supplementary material.

For each primary outcome, a statistically significant result was concluded if the p-value for the trial arm explanatory variable was <0.025.

For peer review only

Sensitivity analyses included repetition of the primary outcome analysis using the ITT population with different missing data models. These included a "best case" (missing set to non-HED), "worst" case (missing set to HED), "conservative case" (missing in control arm set to non-HED, missing in intervention arm set to HED) and multiple imputations (with 50 imputed data sets).

To explore differential intervention effects on the primary measures, pre-specified interaction terms were fitted between trial arm and baseline measures thought to predict the effect of intervention on primary outcomes. These were: age (months) at baseline; gender; socioeconomic status (proportion of students in receipt of FSM tertile split); alcohol use behaviour at baseline – age of initiation, use of alcohol in the year prior to baseline, context of use (abstainer/supervised/unsupervised); and in NI, Grammar/Secondary school.

Process outcomes were assessed across eight pre-specified domains (including intervention acceptability and assessment of the content of EAN), using nine data sources. Methodologies included focus groups with students, an online survey with teachers, and interviews with senior school staff and stakeholders. Fidelity and completeness of delivery were assessed using bespoke tools and calculation of participation rates at the parent/carer evening.

Data cleaning, data management and preliminary analysis were undertaken using IBM SPSS version 20+. Mplus 7.11 was used for all analyses and Stata/IC 12.0 was used to verify Mplus models and generate odds ratios (OR).

For peer review only - http://bmjopen.bmj.com/site/about/guidelines.xhtml

The trial was registered, number ISRCTN47028486.

Ethics approval and consent to participate

The research was approved by Liverpool John Moores University Research Ethics Committee (11/HEA/097). Participants were eligible students in the randomised schools, who consented to participate. Consent was obtained from school head-teachers/principals before randomisation. Consent was obtained from participants and their parents/guardians after randomisation. This was through an opt-out method as opt-in written consent was not required by the ethics committee.

Results

Fig 1 shows participant flow through the trial. School recruitment began in November 2011 and ended in January 2012. As this was a cRCT of an intervention taking place across several years, student numbers refer to those who completed the questionnaire at each data collection period. No participant or parent/carer requested data retrospectively removed from analysis. Multiple data collection ‘mop up’ visits were undertaken with schools, therefore attrition represents students who were absent on data collection days rather than formal drop out. Of the full sample (those who completed a questionnaire at either baseline or 12 months, N=12,738), 10,405 also completed the questionnaire at 33 months (81.7%). There was a higher attrition rate amongst students who were male (19.0%), in receipt of FSM (25.8%), and had used alcohol at baseline (25.4%). There was little difference in attrition between the control and intervention arms of the trial (around one percentage point difference). Attrition also varied by location, with a higher rate in Scotland (24.0%) compared to NI

For peer review only - http://bmjopen.bmj.com/site/about/guidelines.xhtml

(15.0%). Across schools attrition varied from 1.5% to 32.0%. There were no unintended harms or adverse effects reported.

INSERT FIG 1 HERE

Fig 1. School and participant flow diagram - STAMPP Trial. Analysis was conducted at 33 months on students who had completed each of the primary outcome measures. N = number of schools; n = student numbers

Baseline data collection took place in June 2012 with the following follow up data collection points: 12 months (after delivery of phase one of the classroom component); 24 months (after delivery of the parental intervention and phase two of the classroom component); and 33 months. The trial ended as planned after final data collection and analysis.

Baseline characteristics of students (n=11,316) are presented in Table 2. Overall parental/carer participation was low. A total of 319 parent(s)/carer(s) attended the intervention evenings in NI (9% of those eligible) and 63 parents attended in Scotland (2.5%). With respect to the follow-up mailed intervention, 1074 returns were received from parent(s)/carer(s) in NI (a 31% return) and 440 in Scotland (18%).

INSERT TABLE 2 HERE

For peer review only - http://bmjopen.bmj.com/site/about/guidelines.xhtml

Table 2. Baseline characteristics of students according to study condition.

	Control<br>n ($\%_{valid}$)	Intervention<br>n ($\%_{valid}$)
Total (n=11,316)	5567 (49.2)	5749 (50.8)
Gender		
Male	2787 (51.1)	2834 (50.0)
Female	2670 (48.9)	2829 (50.0)
Missing	*110*	*86*
Free School Meals		
No	4289 (77.3)	4436 (77.5)
Yes	1258 (22.7)	1290 (22.5)
Missing	*20*	*23*
Location		
NI	3469 (62.3)	3554 (61.8)
Scotland	2098 (37.7)	2198 (38.2)
Missing	*0*	*0*
HED[a]		
No	5082 (92.2)	5261 (92.4)
Yes	432 (7.8)	431 (7.6)
Missing	*53*	*57*
Ethnicity		
White	4492 (95.3)	4495 (94.5)
Non-white	248 (4.5)	293 (5.5)

For peer review only - http://bmjopen.bmj.com/site/about/guidelines.xhtml

Missing	*827*	*961*

Note: The percentages are calculated on the basis of the complete cases only.

[a] Assessed at baseline as consuming > 5 drinks in one or more episodes in the last 30 days.

Table 3 shows the count and percentages of respondents reporting drinking above the primary outcome threshold (≥6/≥4.5 units) at 33 months, and the adjusted model results by study arm (OR; Incidence rate ratio, IRR). Around one in 5 participants reported at least one episode in the last 30 days. The prevalence of episodes was around nine percentage points higher in the control group (26%) than in the intervention group (17%). Taking the within (pupil) level variance (fixed at 3.29) and the between (school) level variance (0.454 for the full sample), estimated using a null two level model, the corresponding ICC for the full sample was 0.121. Supplementary Table S1 shows the full random intercept models for the primary outcomes at 33 months.

Table 3 Primary outcomes at 33 months by study group

	Unadjusted results		Adjusted model results	
	Control N ($\%_{valid}$)	Intervention N ($\%_{valid}$)	OR/IRR	95% CI
HED (frequency)				
None	3773 (74.4)	4281 (83.0)	0·60	0·49-0·73
One or more occasion	1300 (25.6)	879 (17.0)		
Missing	*1286*	*1219*		
ARH (frequency)				

For peer review only - http://bmjopen.bmj.com/site/about/guidelines.xhtml

None	3126 (60.7)	3408 (65.1)	0·92	0·78-1.05
One or more occasion	2020 (39.3)	1826 (34.9)		
Missing	*1213*	*1145*		
Median (IQR)	0 (2)	0 (3)		

OR, odds ratio; IRR, incidence rate ratio; *HED*, Heavy episodic drinking; ARH, Alcohol related harms

Fig 2 displays the count of respondents reporting ARH at 33 months by study group. Around two thirds of students (63%) reported no alcohol-related harms. The median number of harms was equivalent in each study arm (0), while the interquartile range was smaller in the intervention arm than in the control arm (2 and 3 respectively).

INSERT FIG 2 HERE

Fig 2. Count of school children reporting one or more alcohol related harms by study arm

At the school level, the parameter estimates were significant for the intervention arm (estimate = -0.516, SE=0.102; $p < 0.001$). Schools in the intervention arm had lower levels of HED (their intercepts) than those in the control arm (OR = 0.596, 95% CI 0.490 – 0.725). This represents a significant intervention effect. However, with respect to ARH, the intervention indicator was non-significant suggesting no difference between the intervention and control schools (estimate -0.101, SE = 0.083; p = 0.222; IRR = 0.916, 95% CI 0.780 – 1.052). Identical models were also estimated on the imputed data sets, yielding similar results. For the sensitivity analysis models the intervention arm coefficient remained significant and

For peer review only - http://bmjopen.bmj.com/site/about/guidelines.xhtml

retained the same sign (i.e. being a school in the intervention arm was associated with having a lower intercept), except for the conservative case model.

There were no significant intervention effects observed for primary outcomes assessed at +24 months (Supplementary Table S2); and secondary outcomes assessed at +33 months (Supplementary Table S3) and + 24 months (Supplementary Table S4). Given the high correlation between ever use, last year use and the two primary outcomes assessed at baseline (Supplementary Table S5), subgroup models were estimated on a base of just baseline drinkers (ever and last year use). Whilst the intervention was associated with a significant reduction in the number of self-reported harms amongst baseline drinkers, it did not reduce self-reported harms amongst the non-drinkers at baseline (Supplementary Table S6).

Discussion

In a large cRCT we found that the STAMPP intervention reduced self-reported HED in the past 30 days at 33 months follow-up from baseline, compared with EAN, but not ARH associated with own drinking. There were no clear or consistent effects identified in planned secondary or sub-group analyses (age, gender, SES, alcohol use at baseline, location [Scotland vs NI]). It is possible that longer-term follow-up and/or emphasis on those drinking might reveal such effects, especially with regard to self-reported ARH, which were low in both control and intervention students. The intervention was well received by both pupils and teachers.

For peer review only - http://bmjopen.bmj.com/site/about/guidelines.xhtml

Key strengths of the trial were the large sample size (schools and students), low rates of attrition (no schools dropped out), and relatively high rates of matched data (>80%) across survey waves. This means that the analyses were sufficiently powered. There also appeared to be no comparator bias, as monitoring of delivery of EAN in intervention schools showed that this did not include alcohol education. A major limitation of the work was the failure to attract parents/carers to the brief intervention evening, despite the support of many of the schools. Although all intervention students received a mailed follow up leaflet that reinforced the main messages of the parental intervention, relatively low rates of return of the parental questionnaire suggest that only a minority may have read the mailed information. In contrast, parental participation in the structurally similar (i.e. classroom and parental components) Swedish Örebro Prevention Program, and the Dutch Prevention of Alcohol use in Students (PAS) alcohol prevention programmes were relatively high. (20, 22, 34) Universal interventions such as STAMPP require a range of recruitment strategies as there will be different barriers to, and facilitators of, attendance in parental/carer-based actions. Research is therefore needed to assess the relative efficacy of recruitment strategies such as incentives, mass media campaigns, the removal of barriers to attendance (e.g. providing transport and childcare), and the use of key community recruiters (influential individuals and organisations). (35) Furthermore, it is also important to understand if some parent/carer subgroups (e.g. differentiated on child drinking risk) are more likely to respond to particular recruitment strategies, and if this will lead to recruitment biases.

Although we conducted an ITT analysis which helped to preserve sample size, the achieved participation rates are likely to reflect parental/carer attendance in routine

For peer review only - http://bmjopen.bmj.com/site/about/guidelines.xhtml

UK practice.(36-38) This meant that we were unable to draw any confident inferences about the combined impact of the school and parental intervention (*cf (27)*), or the relative contribution of each component. In practical terms, this means that although the analysis presumed delivery of the combined intervention, discussions with stakeholders about research findings and future delivery are likely to focus on the classroom component (i.e. culturally adapted SHAHRP). However, it is noteworthy that in the PAS programme (20), the classroom component alone did not produce changes in alcohol use behaviours, and these were only observed in pupils receiving the combined intervention. Subsequent mediation analysis of trial data suggested that reduced rate of frequency of drinking or weekly drinking, was mediated by changes in parental rules and attitudes towards alcohol (i.e. more strict rules and attitudes were developed). It is therefore important that similar analyses are undertaken to better understand mediators of behaviour change in STAMPP recipients. Other weaknesses of the study included the lack of blinding in intervention delivery and in some data collectors. It is plausible that lack of blinding in delivery may led to either under- or over-reporting of alcohol use due to social desirability biases, but using an EAN comparator meant that it was not possible to conceal intervention allocation from teachers, who received specialised training and curriculum materials, or pupils, who would typically receive little or no alcohol education in their usual school year. Lack of blinding in some data collectors may have also led to either under- or over-reporting of alcohol use due to social desirability biases, although the use of standardised data collection scripts partly mitigated against this.

Our primary outcome assessment relied on self-report, which may have led to inaccurate reporting of alcohol use through memory, social desirability, and other

For peer review only - http://bmjopen.bmj.com/site/about/guidelines.xhtml

biases.(39) Although adolescent self-reported alcohol questionnaires are generally reliable,(40) there may be differences in reliability between early and late adolescence,(19) and studies of recanting in substance use surveys suggest that this may be an understudied bias in prevention research.(33) However, all students received the same questionnaire and pictorial prompts, and the recall period for the primary outcome used in this study was the previous 30 days, and so if bias had existed, this would have been minimal, and equivalent across trial arms.

Although the classroom component of STAMPP was based on the SHAHRP programme, we did not detect a decrease in ARH. Previous studies of SHAHRP in Australia and NI using quasi-experimental designs found that decreases in self-reported ARH at 32 months were associated with intervention exposure.(21, 28) Differences with the findings of this trial may be related to factors such as methodology, pupil age, changes in the wider drinking culture and public health environment, or other unmeasured cohort effects. Whilst there is a relationship between HED in adolescence and health harms(1) we have planned further exploratory analyses which will investigate ARH, patterns of reporting, and sub group effects in more detail.

Although we are mindful of differences in school autonomy, governance and oversight, and acknowledge regional variability in alcohol use behaviours (e.g.(5)), we believe that the findings of this trial are likely to be generalisable to other geographies. Schools enrolled in the trial were drawn from urban and more rural areas, and from across the socioeconomic gradient. Furthermore, sub group analyses

For peer review only - http://bmjopen.bmj.com/site/about/guidelines.xhtml

showed that there were no differential intervention effects on the basis of school geography (i.e. NI vs Scotland).

Conclusions

The results of this large cRCT provide support for the effectiveness of a combined classroom and brief parental intervention for reducing HED, but not ARH, in young adolescents. Effects on ARH may manifest later, but further research would be required to clarify this.

Acknowledgements

As well as acknowledging the role played by participating schools and school children, the authors would like to acknowledge the support of the following people in this project: Séamus Mullin, Gerry Bleakney, Owen O'Neill (PHANI); Malachy Crudden (CCMS), Maura Kearney, and Fergal Doherty (Psychological Services, Glasgow); Kate Watson (Psychological Services, Inverclyde) and John Butcher and Sandy Cunningham (Education Services, Glasgow). The views and opinions expressed therein are those of the authors and do not necessarily reflect those of the NIHR-PHR, NIHR, NHS or the Department of Health.

A. **Author Contributions**: Sumnall had full access to all of the data in the study and takes responsibility for the integrity of the data and the accuracy of the data analysis. McKay wrote the first draft of the manuscript and subsequent versions; Sumnall was project PI, contributed to the first draft and subsequent iterations of the manuscript, and prepared and submitted the final version of the manuscript; Percy conducted the statistical analysis and contributed to manuscript drafts; Agus,

For peer review only - http://bmjopen.bmj.com/site/about/guidelines.xhtml

Foxcroft, Cole, Murphy, Doherty, Harvey all contributed to drafts and approved the submission.

B. Declaration of interests

No personal competing interests declared. The sponsor University (LJMU) received and administered a payment from the alcohol industry for printing of student workbooks in the Glasgow trial site only. Percy reported that he has previously received funding from the European Foundation of Alcohol Research (ERAB) in relation to the development of statistical models for longitudinal data (2008-2010). Foxcroft reported that his Department has previously received funding from the alcohol industry for unrelated prevention programme training work. Sumnall reported that his Department has previously received funding from the alcohol industry (indirectly via the industry funded Drinkaware charity) for unrelated primary research.

C. Funding

This trial was funded by the National Institute of Health Research (NIHR) Public Health Research (PHR) programme (project number 10/3002/09). The Public Health Agency of NI and Education Boards of Glasgow/Inverclyde provided some intervention costs. Diageo provided funds to print classroom workbooks for use only in the Glasgow Local Authority area. Remaining intervention costs were internally funded. The research and intervention funders had no involvement in intervention design; design and conduct of the study; collection, management, analysis, and interpretation of the data; and preparation, review or approval of the manuscript.

D. Data

For peer review only - http://bmjopen.bmj.com/site/about/guidelines.xhtml

Availability of data and materials: The datasets generated during and/or analysed during the current study are not yet publicly available due to the authors undertaking additional analyses and follow-on studies, but are available from the corresponding author on reasonable request.

References

1. Oesterle S, Hill KG, Hawkins JD, Guo J, Catalano RF, Abbott RD. Adolescent heavy episodic drinking trajectories and health in young adulthood. J Stud Alcohol. 2004;65(2):204-12.

2. Bellis MA, Morleo M, Hughes K, Downing J, Wood S, Smallthwaite L, et al. A cross-sectional survey of compliance with national guidance for alcohol consumption by children: measuring risk factors, protective factors and social norms for excessive and unsupervised drinking. Bmc Public Health. 2010;10:8.

3. McMorris BJ, Catalano RF, Kim MJ, Toumbourou JW, Hemphill SA. Influence of family factors and supervised alcohol use on adolescent alcohol use and harms: similarities between youth in different alcohol policy contexts. J Stud Alcohol Drugs. 2011;72(3):418-28.

4. Livingston JA, Testa M, Hoffman JH, Windle M. Can parents prevent heavy episodic drinking by allowing teens to drink at home? Addict Behav. 2010;35(12):1105-12.

5. Maimaris W, McCambridge J. Age of first drinking and adult alcohol problems: systematic review of prospective cohort studies. J Epidemiol Community Health. 2014;68(3):268-74.

For peer review only

For peer review only - http://bmjopen.bmj.com/site/about/guidelines.xhtml

6. Jones L, McCoy E, Bates G, Bellis MA, Sumnall HR. Understanding the Alcohol Harm Paradox. London: Alcohol Research UK; 2015.

7. Jones L, Bates G, McCoy E, Bellis MA. Relationship between alcohol-attributable disease and socioeconomic status, and the role of alcohol consumption in this relationship: a systematic review and meta-analysis. BMC Public Health. 2015;5.

8. Marmot M. Social determinants of health inequalities. Lancet. 2005;365(9464):1099-104.

9. Foxcroft DR, Tsertsvadze A. Universal family-based prevention programs for alcohol misuse in young people. Cochrane Database Syst Rev. 2011(9):CD009308.

10. Foxcroft DR, Tsertsvadze A. Universal multi-component prevention programs for alcohol misuse in young people. Cochrane Database Syst Rev. 2011(9):CD009307.

11. Foxcroft DR, Tsertsvadze A. Universal school-based prevention programs for alcohol misuse in young people. Cochrane Database Syst Rev. 2011(5):CD009113.

12. Nation M, Crusto C, Wandersman A, Kumpfer KL, Seybolt D, Morrissey-Kane E, et al. What works in prevention. Principles of effective prevention programs. The American psychologist. 2003;58(6-7):449-56.

13. Faggiano F, Vigna-Taglianti FD, Versino E, Zambon A, Borraccino A, Lemma P. School-based prevention for illicit drugs use: a systematic review. Prev Med. 2008;46(5):385-96.

14. Spoth R, Greenberg M, Turrisi R. Preventive interventions addressing underage drinking: state of the evidence and steps toward public health impact. Pediatrics. 2008;121 Suppl 4:S311-36.

For peer review only

For peer review only - http://bmjopen.bmj.com/site/about/guidelines.xhtml

15. Flynn AB, Falco M, Hocini S. Independent evaluation of middle school–based drug prevention curricula: A systematic review. JAMA Pediatrics. 2015;169(11):1046-52.

16. National Institute for Health and Care Excellence. Alcohol: school-based interventions London; 2007.

17. Ryan SM, Jorm AF, Lubman DI. Parenting factors associated with reduced adolescent alcohol use: a systematic review of longitudinal studies. Aust N Z J Psychiatry. 2010;44(9):774-83.

18. Vakalahi HF. Adolescent substance use and family-based risk and protective factors: a literature review. Journal of drug education. 2001;31(1):29-46.

19. Koning IM, Engels RC, Verdurmen JE, Vollebergh WA. Alcohol-specific socialization practices and alcohol use in Dutch early adolescents. J Adolesc. 2010;33(1):93-100.

20. Koning IM, Vollebergh WA, Smit F, Verdurmen JE, Van Den Eijnden RJ, Ter Bogt TF, et al. Preventing heavy alcohol use in adolescents (PAS): cluster randomized trial of a parent and student intervention offered separately and simultaneously. Addiction. 2009;104(10):1669-78.

21. McBride N, Farringdon F, Midford R, Meuleners L, Phillips M. Harm minimization in school drug education: final results of the School Health and Alcohol Harm Reduction Project (SHAHRP). Addiction. 2004;99:278-91.

22. Koutakis N, Stattin H, Kerr M. Reducing youth alcohol drinking through a parent-targeted intervention: the Orebro Prevention Program. Addiction. 2008;103(10):1629-37.

For peer review only - http://bmjopen.bmj.com/site/about/guidelines.xhtml

23. McKay MT, Sumnall HR, Percy A, Cole JC. Self-Esteem and Self-Efficacy: associations with alcohol consumption in a sample of Adolescents in Northern Ireland. Drugs: Education, Prevention, and Policy. 2012;19(1):72-80.

24. McKay MT, Cole JC, Sumnall HR. Teenage Thinking on Teenage Drinking: 15- to 16- year olds' experiences of alcohol in Northern Ireland
Drugs: Education, Prevention, and Policy. 2011;18(5):323-32.

25. McKay MT, Sumnall HR, Goudie AJ, Percy A, Field M, Cole JC. What differentiates Adolescent Problematic Drinkers from their Peers? Results from a cross sectional study in Northern Irish School Children. Drugs: Education, Prevention, and Policy. 2011;18(3):187-99.

26. Farringdon F, McBride N, Midford R. School Health and Alcohol Harm Reduction Project: Formative development of intervention materials and processes. International Journal of Health Promotion and Education. 1999;37(4):137-43.

27. Koning IM, van den Eijnden RJ, Engels RC, Verdurmen JE, Vollebergh WA. Why target early adolescents and parents in alcohol prevention? The mediating effects of self-control, rules and attitudes about alcohol use. Addiction. 2011;106(3):538-46.

28. McKay MT, McBride NT, Sumnall HR, Cole JC. Reducing the harm from adolescent alcohol consumption: results from an adapted version of SHAHRP in Northern Ireland. Journal of Substance Use. 2012;17(2):98-121.

29. Donaldson L. Guidance on the consumption of alcohol by children and young people. London: Department of Health; 2009.

30. van der Vorst H, Engels RC, Meeus W, Dekovic M. The impact of alcohol-specific rules, parental norms about early drinking and parental alcohol use on adolescents' drinking behavior. J Child Psychol Psychiatry. 2006;47(12):1299-306.

For peer review only - http://bmjopen.bmj.com/site/about/guidelines.xhtml

31. Koning IM, Van den Eijnden RJ, Glatz T, Vollebergh WA. Don't Worry! Parental Worries, Alcohol-Specific Parenting and Adolescents' Drinking. Cognitive Therapy Research. 2013;37(1079-1088).

32. McBride N, Midford R, Farringdon F, Phillips M. Early results from a school alcohol harm minimization study: the School Health and Alcohol Harm Reduction Project. Addiction. 2000;95:1021-42.

33. Percy A, McAlister S, Higgins K, McCrystal P, Thornton M. Response consistency in young adolescents' drug use self-reports: A recanting rate analysis. Addiction. 2005;100(2):189-96.

34. Bodin MC, Strandberg AK. The Orebro prevention programme revisited: a cluster-randomized effectiveness trial of programme effects on youth drinking. Addiction. 2011;106(12):2134-43.

35. Segrott J. Recruitment and group composition strategies for family-based substance misuse prevention interventions: an exploratory evaluation. Journal of Children's Services. 2013;8(2):89-109.

36. Caria MP, Faggiano F, Bellocco R, Galanti MR, Group EU-DS. Classroom characteristics and implementation of a substance use prevention curriculum in European countries. Eur J Public Health. 2013;23(6):1088-93.

37. Prinz RJ, Smith EP, Dumas JE, Laughlin JE, White DW, Barron R. Recruitment and retention of participants in prevention trials involving family-based interventions. Am J Prev Med. 2001;20(1 Suppl):31-7.

38. Bauman KE, Ennett ST, Foshee VA, Pemberton M, Hicks K. Correlates of participation in a family-directed tobacco and alcohol prevention program for adolescents. Health Educ Behav. 2001;28(4):440-61.

For peer review only - http://bmjopen.bmj.com/site/about/guidelines.xhtml

39. Leigh BC, Gillmore MR, Morrison DM. Comparison of diary and retrospective measures for recording alcohol consumption and sexual activity. J Clin Epidemiol. 1998;51(2):119-27.

40. Lintonen T, Ahlstrom S, Metso L. The reliability of self-reported drinking in adolescence. Alcohol and alcoholism (Oxford, Oxfordshire). 2004;39(4):362-8.

For peer review only

List of supplementary information table captions

Table S1. Primary outcome alcohol consumption (HED) outcome analysis at 33 months

Table S2 Secondary analysis: primary outcomes at T2

Table S3 Secondary outcomes at T3

Table S4 Secondary outcomes at T2

Table S5 Correlations between baseline alcohol consumption (ever and last year use) and baseline primary outcome indicators (HED and ARH)

Table S6 Summary of intervention effects in primary outcome models (treatment arm parameter estimates only) estimated on baseline drinker and non-drinker sub-groups.

For peer review only - http://bmjopen.bmj.com/site/about/guidelines.xhtml

For peer review only

For peer review only - http://bmjopen.bmj.com/site/about/guidelines.xhtml

Online supplementary material

STAMPP - secondary outcomes and subgroup analyses

Secondary outcomes

A range of secondary outcomes were also examined within the study. These included the primary outcomes assessed at T2:

- *Binge drinking (T2):* Self-reported alcohol use defined as self-reported consumption of >5 drinks, assessed at +24 months (T2) from baseline. This was dichotomised at none/one or more occasions. This outcome was assessed via a two level logistic regression model. Around 12.4% of respondents reported binge drinking at T2 using this measure. In the intervention arm binge drinking was reported by 10.9% (N=573) and in the control arm by 13.9% (N=722).

- *Drinking harms to self (T2):* The number of self-reported harms (harms caused by own drinking) assessed at +24 months (T2) from baseline. Items included harms such as getting into a physical fight or being sick after drinking. The outcome was a count of the number of discrete harms reported (0-16) and was assessed by a two level negative binomial model. In the intervention arm 74.3% reported no drinking harms, while in the control arm 71.5% reported no harms.

In addition, a number of secondary outcomes at T3 and T2 were also examined, including:

For peer review only - http://bmjopen.bmj.com/site/about/guidelines.xhtml

- *Lifetime drinking (T3):* Whether the pupils had ever consumed a full drink of alcohol at +33 months (T3) (two level logistic regression model).

- *Last year drinking (T3):* Whether the pupils had consumed a full drink of alcohol in the last year, assessed at +33 months (T3) (two level logistic regression model).

- *Last month Drinking (T3):* Whether the pupils had consumed a full drink of alcohol in the last month, assessed at +33 months (T3) (two level logistic regression model).

- *Harm from others (T3 and T2):* The number of self-reported harms experienced that were the result of other people's drinking, assessed at both +33 months (T3) and +24 months (T2) from baseline (two level negative binomial models). Harms included being hit or having property damaged by someone who had been drinking.

- *Age of onset (T3 and T2):* Self-reported age at which respondent first consumed a full drink, assessed at both +33 months (T3) and +24 months (T2) from baseline (two level Cox regression model).

- *Unsupervised drinking (T3 and T2):* Whether the pupils were permitted, by their parents(s), to consume alcohol (with small group of friends or at parties) with no adult present, assessed at both +33 months (T3) and +24 months (T2) from baseline (two level logistic regression model).

- *Number of drinks consumed (T3 and T2):* Pupils were asked whether they usually drank from a range of different alcohol drinks (beer, alcopops, spirits cider, wine,

For peer review only

For peer review only - http://bmjopen.bmj.com/site/about/guidelines.xhtml

Buckfast [a popular brand of fortified wine, with caffeine], others) and if so, how much did they usually drink. The values for each drink were summed together to give a total. As the underlying items continued decimals the total value was multiplied by 10 to create whole numbers.

The secondary outcome analysis also included covariates at level 1 (individual) and level 2 (school) where appropriate:

Level 1 covariates

Relevant baseline drinking variable (T0): For each outcome, the corresponding baseline characteristic was included in the model. Mean imputation was used to impute values for those respondents who were missing on this variable. The only model not to include a baseline covariate was age of onset.

Level 2 covariates

Treatment Arm: This was a binary covariate in which schools in the control arm were coded 0 and schools in the intervention arm were coded 1.

Free school meals (Randomisation stratification factor): Schools were classified into three groups based on free school meal provision. The allocation was based on a tertile split based on information provided by head teachers on the proportion of pupils in receipt of free school

For peer review only - http://bmjopen.bmj.com/site/about/guidelines.xhtml

meals: *Low* Free School Meal Provision (0-15.4%), *Moderate* Free School Meal Provision (15.5-30.4%), *High* Free School Meal Provision (30.5% and above).

School type (Randomisation stratification factor): Given the larger number of schools in Northern Ireland, an additional stratification factor was used in the randomisation. This was school type (all boys' school/ all girls' school/coeducation school). Schools in Glasgow/Inverclyde were all assigned to the co-education type. This indicator was used represented by two dummy variables (co-education was the comparison category).

Location: A dummy variable was generated to indicate the location of the schools (Northern Ireland/Scotland).

Analysis of secondary outcomes

Differences in self-reported alcohol use (defined as self-reported consumption of ≥6 units in a single episode in the previous 30 days for males and ≥4.5 units for females - dichotomised at never/one or more occasions) at + 12 months (t1) and +24 months (T2) were assessed using two-level logistic regression models with covariates (baseline alcohol use, sex, SES and location). Similar models were constructed for self-reported alcohol use in lifetime, last year and previous month (all dichotomised) and for unsupervised alcohol use (drinking without the supervision of parents/carers - dichotomised) at +12 months (T1), +24 months (T2) and +33 months (T3).

A negative binomial model with covariates (baseline harms, sex, SES and location) was estimated for the number of self-reported harms (harms caused by own drinking) at +12 months (T1) +24 months (T2). Similar models were estimated for the number of self-reported

For peer review only - http://bmjopen.bmj.com/site/about/guidelines.xhtml

harms caused by the drinking of others and the number of drinks consumed in a 'typical' and the last use episodes at +12 months (T1), +24 months (T2) and +33 months (T3).

Time to alcohol initiation (age at which a whole drink of alcohol was first consumed, not just a sip or a shared drink) at +12 months (T1), +24 months (T2) and +33 months (T3) were compared between trial arms by estimating a two-level Cox proportional hazards model in those who had not already initiated alcohol consumption at baseline. The model controlled for sex, SES and location.

Subgroup analyses

To explore differential treatment effects on the primary and secondary outcome measures, pre-specified interaction terms were fitted between trial arm and baseline measures thought to predict the effect of treatment. These were:

- Age, in months, of pupil at baseline;
- Gender;
- Socioeconomic status (using the proportion of free school meals indicator);
- Alcohol use behaviour at baseline – age of initiation, use of alcohol in the year prior to baseline, context of use (abstainer/supervised/unsupervised);
- and in NI, a Grammar/Secondary school analysis.

For peer review only - http://bmjopen.bmj.com/site/about/guidelines.xhtml

Results

Full primary outcome models

For reasons of space, the full primary outcome model is not presented in the main text. Table S1 presents the random intercept models for the primary outcomes at T3

Table S1. Primary outcome alcohol consumption (HED) outcome analysis at 33 months

	Estimate	S.E.	OR	P value
**ITT Complete case analysis**				
Within level				
Baseline Binge drinking	1.395	0.093	4.036	<0.001
Between Level				
Intervention Arm	-0.516	0.102		<0.001
Free School Meals (tertile)	0.239	0.073		0.001
School Type				
Boys School Dummy	-0.186	0.200		0.35
Girls School Dummy	-0.546	0.266		0.04
Location (NI)	0.422	0.109		<0.001
School level residual variance	0.176	0.035		<0.001
Threshold (BngT3$1)	1.574	0.124		<0.001

Secondary analyses

Results of the secondary analyses are tabulated below. Table S2 presents the random intercept models for the primary outcomes at T2. Results were similar to those found at T3. The baseline measures were significant, as was location. For the binge drinking outcomes both free school meals (tertile split) and school type were significant. The intervention arm was significant at a 0.05 level (β=-0.241; p=0.041). The 2.5% confidence intervals for this parameter ranged from -0.010 to -0.473. However, it failed to reach the much stricter threshold used in the primary analysis (0.025). It should be noted that the binge drinking

For peer review only - http://bmjopen.bmj.com/site/about/guidelines.xhtml

indicator used at T3, and as specified in the DAP, was different that that used at T2. In particular, this measure did not use gender specific splits, referred to drinks rather than units, and did not provide any visual guides to help with the estimation of amount consumed. This suggests that the significant intervention effect may have been partly dependent on the precision of the measurement instrument used to collect the primary outcome data. The age at which differences in binge drinking were assessed may have been important when assessing intervention outcomes.

Table S2 Secondary analysis: primary outcomes at T2

	Estimate	S.E.	OR	P value
**Binge Drinking T2 (ITT CC population logistic model)**				
Within level				
Baseline Binge drinking	1.891	0.101	6.623	<0.001
Between Level				
Treatment Arm	-0.241	0.118		0.04
Free School Meals (tertile)	0.308	0.079		<0.001
School Type				
Boys School Dummy	-0.708	0.297		0.02
Girls School Dummy	-0.608	0.186		0.001
Location	0.732	0.134		<0.001
Residual variance	0.214	0.047		<0.001
Threshold (BngT2$1)	2.698	0.144		<0.001
**Harms to Self T2 (ITT CC population negative binomial model)**				
Within level				
Baseline Harms drinking	0.297	0.016		<0.001
Between Level				
Treatment Arm	-0.144	0.118		0.22
Free School Meals (tertile)	0.162	0.086		0.06
School Type				
Boys School Dummy	-0.247	0.302		0.42
Girls School Dummy	-0.246	0.200		0.22
Location	0.716	0.132		<0.001
Residual variance	0.267	0.054		<0.001
Intercepts (SHarmsT2)	-0.779	0.133		<0.001
Dispersion	4.478	0.304		<0.001

Table S3 presents the outcome models for the secondary outcomes assessed at T3. None of the intervention parameter estimates were significant in these models.

For peer review only

For peer review only - http://bmjopen.bmj.com/site/about/guidelines.xhtml

Table S3 Secondary outcomes at T3

	Estimate	S.E.	OR	P value
**Lifetime drinking T3 (ITT CC population logistic model)**				
Within level				
Baseline Binge drinking	2·070	0·081	7·922	<0·001
Between Level				
Treatment Arm	-0·125	0·102		0·22
Free School Meals (tertile)	0·040	0·070		0·57
School Type				
Boys School Dummy	-0·182	0·209		0·384
Girls School Dummy	-0·501	0·233		0·031
Location	0·597	0·113		<0·001
Residual variance	0·209	0·035		<0·001
Threshold (LifeT3$1)	0·419	0·114		<0·001
**Last year drinking T3 (ITT CC population logistic model)**				
Within level				
Baseline Last year drinking	1·822	0·086	6·187	<0·001
Between Level				
Treatment Arm	-0·126	0·096		0·19
Free School Meals (tertile)	0·011	0·065		0·87
School Type				
Boys School Dummy	-0·176	0·211		0·40
Girls School Dummy	-0·401	0·229		0·08
Location	0·615	0·105		<0·001
Residual variances	0·177	0·032		<0·001
Threshold (LYearT3$1)	0·485	0·103		<0·001
**Last month drinking T3 (ITT CC population logistic model)**				
Within level				
Baseline Last month drinking	1·329	0·114	3·779	<0·001
Between Level				
Treatment Arm	-0·149	0·094		0·11
Free School Meals (tertile)	0·114	0·069		0·10
School Type				
Boys School Dummy	-0·333	0·213		0·12
Girls School Dummy	-0·330	0·237		0·16
Location	0·381	0·104		<0·001
Residual variances	0·148	0·028		<0·001
Threshold (LMonthT3$1)	1·459	0·102		<0·001
**Harms from others drinking T3 (ITT CC population NB model)**				
Within level				
Baseline Harms (others)	0·330	0·016		<0·001
Between Level				
Treatment Arm	0·000	0·057		0·10
Free School Meals (tertile)	0·077	0·042		0·07
School Type				
Boys School Dummy	0·117	0·116		0·31
Girls School Dummy	-0·070	0·172		0·68
Location	0·167	0·063		0·01
Residual variance	0·050	0·014		<0·001
Dispersion	1·301	0·071		<0·001
Intercept	-0·733	0·061		<0·001
**Age of onset T3 (ITT CC population Cox regression model)**				
Between Level				
Treatment Arm	-0·095	0·067		0·16
Free School Meals (tertile)	0·054	0·047		0·25

For peer review only

For peer review only - http://bmjopen.bmj.com/site/about/guidelines.xhtml

	Estimate	S.E.	P value
School Type			
Boys School Dummy	-0·299	0·146	0·04
Girls School Dummy	-0·407	0·145	0·01
Location	0·344	0·075	<0·001
Residual variance	0·097	0·017	< 0·001

Table S4 presents the models for the secondary outcomes assessed at T2. Again, none of the intervention parameter estimates were significant in these models.

Table S4 Secondary outcomes at T2

	Estimate	S.E.	P value
**Harms from others drinking T2 (ITT CC population NB model)**			
Within level			
Baseline Harms (others)	0·421	0.017	<0.001
Between Level			
Treatment Arm	-0·058	0.060	0.33
Free School Meals (tertile)	0·132	0.044	0.003
School Type			
Boys School Dummy	0·144	0.108	0.18
Girls School Dummy	0·075	0.119	0.53
Location	0·255	0.071	<0.001
Residual variance	0·058	0.011	<0.001
Dispersion	1·032	0.078	<0.001
Intercept	-1·079	0.069	<0.001
**Age of onset T2 (ITT CC population Cox regression model)**			
Between Level			
Treatment Arm	-0·055	0.074	0.46
Free School Meals (tertile)	0·084	0.048	0.08
School Type			
Boys School Dummy	-0·528	0.197	0.007
Girls School Dummy	-0·453	0.169	0.007
Location	0·408	0.083	<0.001
Residual variance	0·176	0.028	<0.01
**Unsupervised drinking T2 (ITT CC population Logistic model)**			
Within level			
Baseline unsupervised drinking	2·114	0.097	<0.001
Between Level			
Treatment Arm	-0·087	0.100	0.39
Free School Meals (tertile)	0·166	0.066	0.01
School Type			
Boys School Dummy	-0·306	0.217	0.16
Girls School Dummy	-0·207	0.135	0.12
Location	0·669	0.112	<0.001
Residual variance	0·170	0.038	<0.001
Threshold (Unsuper$1)	1·883	0.118	<0.001
**Number of drinks T2 (ITT CC population NB model)**			
Within level			
Baseline unsupervised	0·170	0.013	<0.001
Between Level			
Treatment Arm	-0·088	0.096	0.36
Free School Meals (tertile)	0·125	0.068	0.07
School Type			
Boys School Dummy	-0·574	0.259	0.03

For peer review only

For peer review only - http://bmjopen.bmj.com/site/about/guidelines.xhtml

Girls School Dummy	-0·181	0.147	0.22
Location	0·583	0.105	<0.001
Residual variances	0·153	0.035	<0.001
Intercept (NumDrkT2)	2·836	0.106	<0.001
Dispersion (NumDrkT2)	5·671	0.340	<0.001

Subgroup analyses

To explore differential treatment effects on the primary measures interaction terms were fitted between trial arm and baseline measures thought to predict the effect of treatment. Initial pre-specified subgroup analysis examined baseline alcohol consumption (ever use, last year use, age of onset, unsupervised drinking). Given the high correlations between ever use, last year use and the two primary outcomes assessed at baseline (binge drinking and alcohol harms) (see Table), subgroup models were estimated on a base of just baseline drinkers (ever and last year use) to examine the possibility of the intervention having a differential impact on drinkers compared to non-drinkers at baseline.

Table S5 Correlations between baseline alcohol consumption (ever and last year use) and baseline primary outcome indicators (HED and ARH)

	Ever use (T0)	Last year use (T0)
HED (BngT0)	0·426	0·434
ARH (harmsT0)	0·506	0·515

For HED, the treatment arm was significant in both the drinker only models (both last year and ever use) and the corresponding non-drinker only models (

Table). This means that no differential intervention effect on binge drinking, dependent on baseline drinking, was detected. However, for ARH, whilst the intervention was associated with a significant reduction in the number of self-reported harms amongst drinkers (either defined as ever or last year use at baseline), it did not reduce self-reported harms amongst the non-drinkers at baseline. When the ever use and last year use subgroup effects were

For peer review only

examined via interaction terms (on the full CC population) the interaction terms for harms were non-significant, as were the interaction terms for age of onset and unsupervised drinking.

Table S6 Summary of intervention effects in primary outcome models (treatment arm parameter estimates only) estimated on baseline drinker and non-drinker sub-groups.

	N	Estimate	S.E.	P value
**Binge drinking primary outcome models**				
1. Treatment arm (*Limited to pupils reporting ever used alcohol at T0*)	2011	-0·504	0·127	<0·001
2. Treatment arm (*Limited to pupils reporting never used alcohol at T0*)	7145	-0·570	0·123	<0·001
3. Treatment arm (*Limited to pupils reporting used in last year at T0*)	1617	-0·484	0·141	0·001
4. Treatment arm (*Limited to pupils reporting didn't use in last year at T0*)	7512	-0·582	0·118	<0·001
**Harms primary outcomes models**				
1. Treatment arm (*Limited to pupils reporting ever used alcohol at T0*)	2053	-0·145	0·054	0·008
2. Treatment arm (*Limited to pupils reporting never used alcohol at T0*)	7233	-0·094	0·097	0·330
3. Treatment arm (*Limited to pupils reporting used in last year at T0*)	1644	-0·127	0·058	0·028
4. Treatment arm (*Limited to pupils reporting didn't use in last year at T0*)	7615	-0·069	0·096	0·314

Note: The primary outcome models summarised here were identical to the primary outcome model outlined above except for being restricted to just the subgroup members (drinkers and non-drinkers)

In the additional pre-specified subgroup analysis model estimated (age, gender), the corresponding interaction terms were all non-significant.

For peer review only

Table S1: CONSORT 2010 checklist of information to include when reporting a cluster randomised trial

Section/Topic	Item No	Standard Checklist item	Extension for cluster designs	Page No *
**Title and abstract**				
	1a	Identification as a randomised trial in the title	Identification as a cluster randomised trial in the title	1
	1b	Structured summary of trial design, methods, results, and conclusions (for specific guidance see CONSORT for abstracts)[1,2]	See table 2	2
**Introduction**				4
**Background and objectives**	2a	Scientific background and explanation of rationale	Rationale for using a cluster design	6
	2b	Specific objectives or hypotheses	Whether objectives pertain to the the cluster level, the individual participant level or both	5, 11
**Methods**				6
**Trial design**	3a	Description of trial design (such as parallel, factorial) including allocation ratio	Definition of cluster and description of how the design features apply to the clusters	6
	3b	Important changes to methods after trial commencement (such as eligibility criteria), with reasons		N/A
**Participants**	4a	Eligibility criteria for participants	Eligibility criteria for clusters	6-7
	4b	Settings and locations where the data were collected		6
**Interventions**	5	The interventions for each group with sufficient details to allow replication, including how and when they were actually administered	Whether interventions pertain to the cluster level, the individual participant level or both	8 & Table 1
**Outcomes**	6a	Completely defined pre-specified primary and secondary outcome measures, including how and	Whether outcome measures pertain to the cluster level, the individual participant level or both	11

For peer review only

		when they were assessed		
	6b	Any changes to trial outcomes after the trial commenced, with reasons		12
**Sample size**	7a	How sample size was determined	Method of calculation, number of clusters(s) (and whether equal or unequal cluster sizes are assumed), cluster size, a coefficient of intracluster correlation (ICC or *k*), and an indication of its uncertainty	12
	7b	When applicable, explanation of any interim analyses and stopping guidelines		**N/A**
**Randomisation:**				
**Sequence generation**	8a	Method used to generate the random allocation sequence		**7**
	8b	Type of randomisation; details of any restriction (such as blocking and block size)	Details of stratification or matching if used	7
**Allocation concealment mechanism**	9	Mechanism used to implement the random allocation sequence (such as sequentially numbered containers), describing any steps taken to conceal the sequence until interventions were assigned	Specification that allocation was based on clusters rather than individuals and whether allocation concealment (if any) was at the cluster level, the individual participant level or both	7
**Implementation**	10	Who generated the random allocation sequence, who enrolled participants, and who assigned participants to interventions	Replace by 10a, 10b and 10c	7
	10a		Who generated the random allocation sequence, who enrolled clusters, and who assigned clusters to interventions	6
	10b		Mechanism by which individual participants were included in clusters for the purposes of the trial (such as complete	7

For peer review only

			enumeration, random sampling)	
	10c		From whom consent was sought (representatives of the cluster, or individual cluster members, or both), and whether consent was sought before or after randomisation	7
**Blinding**	11a	If done, who was blinded after assignment to interventions (for example, participants, care providers, those assessing outcomes) and how		**7-8**
	11b	If relevant, description of the similarity of interventions		**11**
**Statistical methods**	12a	Statistical methods used to compare groups for primary and secondary outcomes	How clustering was taken into account	12
	12b	Methods for additional analyses, such as subgroup analyses and adjusted analyses		**14**
**Results**				15
**Participant flow (a diagram is strongly recommended)**	13a	For each group, the numbers of participants who were randomly assigned, received intended treatment, and were analysed for the primary outcome	For each group, the numbers of clusters that were randomly assigned, received intended treatment, and were analysed for the primary outcome	15 & Figure 1
	13b	For each group, losses and exclusions after randomisation, together with reasons	For each group, losses and exclusions for both clusters and individual cluster members	15 & Figure 1
**Recruitment**	14a	Dates defining the periods of recruitment and follow-up		**11**
	14b	Why the trial ended or was stopped		**16**
**Baseline data**	15	A table showing baseline demographic and clinical	Baseline characteristics for the individual and cluster levels as	15 & Table 2

For peer review only

		characteristics for each group	applicable for each group	
**Numbers analysed**	16	For each group, number of participants (denominator) included in each analysis and whether the analysis was by original assigned groups	For each group, number of clusters included in each analysis	Table 2
**Outcomes and estimation**	17a	For each primary and secondary outcome, results for each group, and the estimated effect size and its precision (such as 95% confidence interval)	Results at the individual or cluster level as applicable and a coefficient of intracluster correlation (ICC or k) for each primary outcome	18
	17b	For binary outcomes, presentation of both absolute and relative effect sizes is recommended		**18-19**
**Ancillary analyses**	18	Results of any other analyses performed, including subgroup analyses and adjusted analyses, distinguishing pre-specified from exploratory		**20 & online supplementary material**
**Harms**	19	All important harms or unintended effects in each group (for specific guidance see CONSORT for harms[3])		**15**
**Discussion**				20
**Limitations**	20	Trial limitations, addressing sources of potential bias, imprecision, and, if relevant, multiplicity of analyses		**21-22**
**Generalisability**	21	Generalisability (external validity, applicability) of the trial findings	Generalisability to clusters and/or individual participants (as relevant)	23
**Interpretation**	22	Interpretation consistent with results, balancing benefits and harms, and considering other relevant evidence		**23-24**
**Other information**				
**Registration**	23	Registration number and		**14**

For peer review only - http://bmjopen.bmj.com/site/about/guidelines.xhtml

		name of trial registry	
**Protocol**	24	Where the full trial protocol can be accessed, if available	**6**
**Funding**	25	Sources of funding and other support (such as supply of drugs), role of funders	**24 and information included as part of journal submission process**

** Note: page numbers optional depending on journal requirements*

For peer review only

Table 2: Extension of CONSORT for abstracts[1,2] to reports of cluster randomised trials

Item	Standard Checklist item	Extension for cluster trials
**Title**	Identification of study as randomised	**Identification of study as cluster randomised**
**Trial design**	Description of the trial design (e.g. parallel, cluster, non-inferiority)	
**Methods**		
**Participants**	Eligibility criteria for participants and the settings where the data were collected	**Eligibility criteria for clusters**
**Interventions**	Interventions intended for each group	
**Objective**	Specific objective or hypothesis	**Whether objective or hypothesis pertains to the cluster level, the individual participant level or both**
**Outcome**	Clearly defined primary outcome for this report	**Whether the primary outcome pertains to the cluster level, the individual participant level or both**
**Randomization**	How participants were allocated to interventions	**How clusters were allocated to interventions**
**Blinding (masking)**	Whether or not participants, care givers, and those assessing the outcomes were blinded to group assignment	
**Results**		
**Numbers randomized**	Number of participants randomized to each group	**Number of clusters randomized to each group**
**Recruitment**	Trial status[1]	
**Numbers analysed**	Number of participants analysed in each group	**Number of clusters analysed in each group**
**Outcome**	For the primary outcome, a result for each group and the estimated effect size and its precision	**Results at the cluster or individual participant level as applicable for each primary outcome**
**Harms**	Important adverse events or side effects	
**Conclusions**	General interpretation of the results	
**Trial registration**	Registration number and name of trial register	
**Funding**	Source of funding	

[1] Relevant to Conference Abstracts

REFERENCES

[1] Hopewell S, Clarke M, Moher D, Wager E, Middleton P, Altman DG, et al. CONSORT for reporting randomised trials in journal and conference abstracts. *Lancet* 2008, 371:281-283

[2] Hopewell S, Clarke M, Moher D, Wager E, Middleton P, Altman DG at al (2008) CONSORT for reporting randomized controlled trials in journal and conference abstracts: explanation and elaboration. *PLoS Med* 5(1): e20

[3] Ioannidis JP, Evans SJ, Gotzsche PC, O'Neill RT, Altman DG, Schulz K, Moher D. Better reporting of harms in randomized trials: an extension of the CONSORT statement. *Ann Intern Med* 2004; 141(10):781-788.

For peer review only

BMJ Open

Steps towards alcohol misuse prevention programme (STAMPP): a school and community based cluster randomised controlled trial

Journal:	*BMJ Open*
Manuscript ID	bmjopen-2017-019722.R1
Article Type:	Research
Date Submitted by the Author:	19-Dec-2017
Complete List of Authors:	McKay, Michael; Psychological Sciences<br>Agus, Ashley; Northern Ireland Clinical Trials Unit<br>Cole, Jonathan; University of Liverpool, Psychological Sciences<br>Doherty, Paul; Northern Ireland Clinical Trials Unit<br>Foxcroft, David; Oxford Brookes University UK<br>Harvey, Séamus; School of Sport, Health, and Exercise Sciences<br>Murphy, Lynn; Northern Ireland Clinical Trials Unit<br>Percy, Andrew; Queens University , School of Social Sciences, Education, and Social Work<br>Sumnall, Harry; Liverpool John Moores University, Public Health Institute
**Primary Subject Heading**:	Public health
Secondary Subject Heading:	Addiction, Evidence based practice, Public health
Keywords:	alcohol, school based intervention, prevention, alcohol related harm, universal prevention, adolescents

SCHOLARONE™
Manuscripts

Steps towards alcohol misuse prevention programme (STAMPP): a school and community based cluster randomised controlled trial

Corresponding author: Michael McKay[1 #3];

Email: Michael.McKay@liverpool.ac.uk

Tel: 0044 7875778186

Authors:

Michael McKay [1, #3] (Michael.McKay@liverpool.ac.uk), Ashley Agus [2] (ashley.agus@nictu.hscni.net); Jon Cole [3] (j.c.cole@liv.ac.uk); Paul Doherty [2] (paul.doherty@nictu.hscni.net); David R. Foxcroft [4] (david.foxcroft@brookes.ac.uk); Séamus Harvey [1,#6] (harveyseamus@gmail.com); Lynn Murphy [2] (lynn.murphy@nictu.hscni.net); Andrew Percy [5] (a.percy@qub.ac.uk); Harry Sumnall [1] (h.sumnall@ljmu.ac.uk).

[1] Public Health Institute, Liverpool John Moores University, UK

[2] Northern Ireland Clinical Trials Unit, The Royal Hospitals, Belfast, UK

[3] Department of Psychological Sciences, University of Liverpool, UK

[4] Psychology and Public Health, Oxford Brookes University, UK

[5] School of Social Sciences, Education, and Social Work, Queen's University Belfast, UK

[6] School of Sport, Health and Exercise Sciences, University of Bangor, UK

# Current address

For peer review only - http://bmjopen.bmj.com/site/about/guidelines.xhtml

Abstract (Word count: 296)

Objectives: To assess the effectiveness of a combined classroom curriculum and parental intervention (The Steps Towards Alcohol Misuse Prevention Programme; STAMPP), compared to alcohol education as normal (EAN), in reducing self-reported heavy episodic drinking (HED) and alcohol-related harms (ARH) in adolescents.

Setting: 105 High schools in Northern Ireland (NI) and in Scotland.

Participants: Schools were stratified by free school meal provision. Schools in NI were also stratified by school type (male/female/co-educational). Eligible students were in school year 8/S1 (aged 11-12) at baseline (June 2012).

Intervention: A classroom-based alcohol education intervention, coupled with a brief alcohol intervention for parents/carers.

Primary Outcomes: (i) the prevalence of self-reported HED in the previous 30 days, and (ii) the number of self-reported ARHs in the previous six months. Outcomes were assessed using two level random intercepts models (logistic regression for HED and negative binomial for number of ARHs).

Results: At 33 months data were available for 5,160 intervention and 5,073 control students (HED outcome), and 5,234 and 5,146 students (ARH outcome) respectively. Of those who completed a questionnaire at either baseline or 12 months (N=12,738), 10,405 also completed the questionnaire at 33 months (81.7%). Fewer students in the Intervention group reported HED compared to EAN (17% versus 26%; odds

For peer review only

For peer review only - http://bmjopen.bmj.com/site/about/guidelines.xhtml

ratio=0·60, 95% CI 0·49-0·73), with no significant difference in the number of self-reported ARHs (incident rate ratio = 0·92, CI 0·78-1.05). Although the classroom component was largely delivered as intended, there was low uptake of the parental component. There were no reported adverse effects.

Conclusions: Results suggest that STAMPP could be an effective programme to reduce HED prevalence. Whilst there was no significant reduction in ARH, it is plausible that effects on harms would manifest later.

Trial Registration: The date of trial registration (ISRCTN47028486 (http://www.isrctn.com/ISRCTN47028486) was 23/09/2011, and school recruitment began 01/11/2011.

For peer review only

Article Summary

Strengths and Limitations.

- All data are longitudinal;
- The sample size was very large and attrition relatively low;
- Schools were independently randomised;
- Some of those involved in fieldwork were not blind to participant condition;
- Overall levels of alcohol-related harm were low.

Keywords: alcohol; prevention; school based intervention; alcohol related harm; universal prevention; adolescents

For peer review only - http://bmjopen.bmj.com/site/about/guidelines.xhtml

Introduction

Adolescence is a period when young people experiment with alcohol, and as they age the amount and frequency of consumption increases.(1) Research has shown that family socialisation factors such as approval of adolescent drinking and the provision of alcohol in the home predicts drinking among adolescents and young adults (2-4) An earlier onset of self-reported drunkenness and the establishment of regular alcohol drinking is associated with a greater risk of alcohol-related problems in adulthood.(5) There are also clear geographic and socioeconomic differences in the burden alcohol places on the population, and these are closely associated with other major indicators of ill health and health inequalities.(6-8)

Previous literature reviews have highlighted a lack of high quality trials of universal school-based alcohol prevention programmes, and few approaches studied have shown positive intervention effects.(9-15) However, while reviews have been unable to recommend any single prevention initiative, many have concluded that interventions that develop social skills appear to be superior to those that seek to enhance only knowledge.(10-13) Guidance issued by the National Institute for Health and Care Excellence (NICE) in the UK in 2007 called for partnerships between schools and other stakeholders in efforts to prevent misuse.(16) Reviews of universal alcohol prevention in family settings suggest that activities supporting parenting skills, including establishing clear boundaries or rules and parental monitoring, may be effective.(9, 17-19) Primary studies also suggest that when combined with a school-based alcohol curriculum, provision of advice to parents about setting strict rules around alcohol consumption reduces adolescent drinking.(20, 21) Indeed a

For peer review only - http://bmjopen.bmj.com/site/about/guidelines.xhtml

recently-published systematic review reported that of ten identified combined child- and parent-based interventions, nine had reported significant and lasting positive effects on adolescent substance use (22).

The Steps Towards Alcohol Misuse Prevention Programme (STAMPP) intervention combined a culturally adapted intervention based on the School Health and Alcohol Harm Reduction Project (SHAHRP)(23) curriculum with a researcher-developed brief parental intervention based on the Swedish Örebro Prevention Program.(24) SHAHRP is an example of a resistance skills training programme, and includes elements of alcohol-specific personal and social skills training.(25-28) In accordance with the theoretical assumptions underlying such programmes, it includes three main strategies: (i) teaching students to recognise high-risk situations, (ii) increasing the awareness of external influences on behaviour, and (iii) combining self-control (i.e. the ability to control responses, to interrupt undesired behavioural tendencies and refrain from acting upon them) with refusal skills training (i.e. in order to improve self-efficacy in avoiding unhealthy behaviours, but not with the consequence of social disadvantage for the young person with their peers). The knowledge delivered through SHAHRP (e.g. lessons on effects of alcohol, description of alcohol units) was not assumed to have direct preventative effects, but instead hypothesised to shape alcohol attitudes and support situation-specific decision making. The parental component was based on research indicating that restrictive parenting practices (e.g., monitoring of children's alcohol use, healthy attitudes towards alcohol, alcohol rule-setting) was associated with reduced prevalence of children's alcohol use (21). When this approach was delivered alongside a classroom intervention in the Dutch Prevention of Alcohol Use in Students, programme effect

For peer review only - http://bmjopen.bmj.com/site/about/guidelines.xhtml

was mediated through children's perceptions of parental rules, child self-efficacy, and child self-control.(29)

It was hypothesised that fewer students in schools delivering STAMPP would self-report: (i) past 30-day heavy episodic drinking (HED) at final follow-up (33 months from baseline); and (ii) fewer self-reported alcohol-related harms (ARH) at final follow-up than those in schools delivering alcohol education as normal (EAN). These primary aim of the research trial were to assess whether STAMPP was effective in reducing self-reporting of these two indicators of alcohol misuse.

Materials and Methods

Study design

This was a cluster randomised controlled trial (cRCT) of school children in Northern Ireland (NI) and Glasgow/Inverclyde Education Authority (Scotland) areas in the United Kingdom (UK) with schools as the unit of randomisation. The research was approved by Liverpool John Moores University Research Ethics Committee (11/HEA/097). The trial protocol is available from http://www.nets.nihr.ac.uk/projects/phr/10300209.

Participants

The sampling frame comprised all mainstream post primary schools in NI (excluding those within the Eastern Health Board due to existing delivery of SHAHRP in that area) and in Glasgow/Inverclyde Local Authorities. All schools in the sampling frame

For peer review only - http://bmjopen.bmj.com/site/about/guidelines.xhtml

were assessed for satisfaction of the inclusion criteria and willingness to participate in the trial.

A total of 105 schools were invited to participate in the trial, and all accepted; 70 in NI, 30 in Glasgow Local Authority and five in Inverclyde Local Authority. Inclusion criteria were schools in NI and Scotland that taught students in school year 8/S1 in the academic year 2011/2012 (aged 11/12 at randomisation). Exclusion criteria were schools that did not include students in the specified school year, or only provided non-mainstream or vocational education (e.g. pupil referral units, further education colleges). Individual students with special educational needs in mainstream classrooms were excluded at the discretion of teachers as the intervention materials had not been developed for use with this population.

Participants were eligible students in the randomised schools, who consented to participate. Opt in consent was obtained from school head-teachers/principals before randomisation. Opt out consent from participants and their parents/guardians was obtained after randomisation. No schools withdrew from the trial and no pupils or parents/carers withdrew consent. Data was collected under examination-like conditions on school premises.

Randomisation and blinding

Schools were randomly assigned (1:1) to receive STAMPP or alcohol EAN before baseline data were collected. Randomisation was performed by an independent statistician blinded to the identity of the schools. All schools were stratified on Free School Meal Provision (FSM; low/moderate/high), which was taken as a proxy for

For peer review only - http://bmjopen.bmj.com/site/about/guidelines.xhtml

socio-economic status. Schools in NI were also stratified by school-type (male/female/co-educational).

Schools, students, intervention trainers and delivery staff (teachers) were not blinded to study condition. Data collection was undertaken by a team of researchers that included the trial manager and research assistants, some of whom were not blinded to study condition.

Data analysis of primary and secondary outcomes was undertaken by the trial statistician who was blinded to the study condition.

Procedures

STAMPP combined a school-based skills development curriculum, and a brief parental intervention designed to support parents in setting family rules around drinking (see Table 1 for overview of the intervention). The classroom component of STAMPP was based on the SHAHRP intervention and culturally adapted for the settings of delivery.(30) It combined skills training, education, and activities designed to encourage positive behavioural change.(23) See supplementary materials for more details on the content of each lesson. It was a curriculum-based programme delivered in two phases over a two year period. As part of the trial, the first phase was delivered when students were in school year 9/S2 (age 12-13 years) and the second phase was delivered during the subsequent year.

The parental component of STAMPP was developed by the trial team and was based on the programme structure of Koutakis and colleagues (24), and Koning and

For peer review only - http://bmjopen.bmj.com/site/about/guidelines.xhtml

colleagues. (20, 21) The component differed in two main ways to these earlier programmes. Firstly, as part of STAMPP, delivery of a single parental component coincided with the delivery of phase two of the classroom curriculum, whereas in Koutakis and Koning, parents' evenings were held several times over the intervention delivery phase. Secondly, the session was partly based upon guidelines included in the UK Chief Medical Officers' 2009 guidelines for drinking in childhood (31). All intervention pupil parents, regardless of whether they had attended the evening or not, were mailed an information leaflet a few weeks after the parental session which reinforced the discussion points.

For peer review only

Table 1. Stages in the STAMPP Trial

Stage	Description
Recruitment of schools	• Schools in Glasgow Local Authority (n = 30) were recruited as a complete group following negotiations with Education Services<br>• Schools in Inverclyde (n = 5) were recruited following a meeting with the Headteachers/Principals to discuss the practicalities of the trial.<br>• Schools in Northern Ireland (n = 70) were recruited individually in the following process: letter of information; follow-up telephone call; individual meeting with Headteacher/ Principals; agree yes/no.
Training of teachers	• One-day training events were held in each study site before both phases of delivery of the classroom component. Training for the following academic year (from September onwards) took place in the preceding June.<br>• Training involved lectures on alcohol (e.g. effects of alcohol use; prevalence rates; risk and protective factors for alcohol use), sharing experiences on previous delivery of the programme, and skills rehearsal for each of the SHAHRP lessons.<br>• Training involved examination of each of the SHAHRP Lessons which covered: Myths about Alcohol; Units of Alcohol; Reasons why people do/don't drink; Alcohol and the Body; Consequences of 'levels' of drinking; Blood Alcohol Concentration; Social and Personal Harms; Alcohol Policy; Alcohol and the Media; Advice for Teenagers; A 'Night Out'; Pressures faced by Young Drinkers; Scenario-based discussion.<br>• Each lesson was scheduled to last one lesson period (approximately 40 minutes) and delivered once a week

For peer review only

For peer review only - http://bmjopen.bmj.com/site/about/guidelines.xhtml

	• Teachers were provided with support materials (CD-ROMS, workbooks) at each training session to help implement the lessons.
Intervention Period	• The intervention period was September to November in both academic years. Phase One involved six lessons and Phase Two, four lessons. Schools were asked to complete all lessons within the three-month delivery window in both phases.<br>• The Parental Brief Intervention coincided with delivery of Phase Two when the children were in their third year of secondary school and took place in the evening on Intervention school premises The intervention included a brief presentation on the UK Chief Medical Officers' guidelines on alcohol use by young people, and a discussion on setting family rules on alcohol. All Intervention student parents, regardless of whether they had attended the evening or not, were mailed a leaflet which reinforced these points a few weeks after the parental session<br>• Final data collection for the primary outcome took place one year after all elements of the intervention had been delivered.

For peer review only - http://bmjopen.bmj.com/site/about/guidelines.xhtml

For peer review only

The control group participants continued with alcohol EAN within their school. In NI, alcohol-related education is delivered in the context of the Personal Development dimension of Learning for Life and Work (32) while in Scotland, alcohol education is delivered within the context of Curriculum for Excellence (33). In both contexts guidelines are offered to schools, however, the precise nature and duration of EAN is at the discretion of individual school Managers. Parents/carers of control students did not receive the STAMPP intervention or materials, but may have been exposed to alcohol intervention activities in the community as part of independent provision.

Questionnaires were administered to participants at baseline in June 2012 and at three follow-ups: +12, +24, and +33 months. All students that were present at baseline or joined participating schools prior to delivery of Phase 1 of the intervention were included in the analyses. Parents/carers were asked to complete a short postal questionnaire, which coincided with delivery of the information leaflet. Alcohol rules were assessed using a 10-item scale to measuring the degree to which parents/carers permitted their children to consume alcohol in various situations, such as 'in the absence of parents at home' or 'at a friend's party' ($\alpha = 0.86 - 0.90$). (34) Parental alcohol self-efficacy was assessed using a three item scale assessing the level of confidence the parent/carer had in their own ability to prevent their child from drinking ($\alpha = 0.67$).(35) This data was collected to inform future mediation analysis and is not reported here.

Outcomes

For peer review only - http://bmjopen.bmj.com/site/about/guidelines.xhtml

The study had two primary outcomes at 33 months; (i) the prevalence of self-reported HED drinking in the previous 30 days (HED defined as the consumption of ≥6 units [males]/ ≥4.5 units [females] on one or more occasions) and (ii) the number of self-reported harms (caused by own drinking) in the previous six months in students. Pre-specified secondary outcomes are described in the online supplementary material, except for those related to the cost-effectiveness analysis which will be reported elsewhere. The original primary outcome was self-reported frequency of consumption of >5 'drinks' in a single drinking episode. However, concerns arose because it became clear that >5 'drinks' could refer to drinks of different alcohol strength and volume. As the objective of the intervention was to reduce HED, the primary outcome was changed to consumption of ≥6 units for males, and ≥4.5 units for females – both are 1.5 times the Chief Medical Officer's maximum daily guideline for adults,(31) and this was ratified by the independent Study Steering Committee. This change was implemented before the final wave of data collection, before unblinding, and before any analysis of trial outcome measures at any data collection point had been undertaken.

To assess the HED primary outcome, participants were presented with pictorial prompts of how much alcohol ≥6/≥4.5 UK units represents. Pictures presented the most popular drinks consumed in the two study areas and respondents were asked to report the frequency of consuming this amount of alcohol over the previous month. Harms associated with own use of alcohol were measured using a 16-item scale developed for the Australian SHAHRP trial (internal consistency 0.9).(36) Participants were asked to indicate on a Likert scale how many times in the past six months they had experienced the individual harm. For example, participants were

For peer review only - http://bmjopen.bmj.com/site/about/guidelines.xhtml

asked to report frequency of having a hangover after drinking, or if they had got into a physical fight when drinking.

Statistical analysis

It was calculated that a sample size of 90 schools (45 per study arm; 80 students per school) would be powerful enough (80%; α= 0.05; ICC = 0.09 based on data from the Belfast Youth Development Study (37)) to detect a standardised effect size of δ = 0.2, or a 10% absolute reduction in risk (51% vs. 41%) for the primary outcome of HED. Assuming 20% attrition within each cluster (from 100 to 80 students), the target sample size was 90 schools and 9000 students at baseline.

Summary statistics on school and student recruitment, withdrawal and dropout were collated for both trial arms and reported as a participant flow diagram for reporting of cRCT (Fig 1). Outcome measure scores from the questionnaires were summarised and tabulated for the trial arms.

The outcome analysis was an Intention to Treat (ITT) analysis using the Complete Case (CC) population such that all cases were assessed regardless of intervention and intervention dosage. Logistic regression models estimated the association between STAMPP and the odds of self-reported HED. Negative binomial regression models estimated the association between STAMPP and the number of ARH. All models included school-level random intercepts to account for correlation due to clustering of students within schools. All models adjusted for factors used to stratify randomization and the outcome's corresponding value at baseline. For details of analysis of secondary outcomes please see the supplementary material. For each

For peer review only - http://bmjopen.bmj.com/site/about/guidelines.xhtml

primary and secondary outcome, a statistically significant result was concluded if the *p*-value for the treatment arm explanatory variable was <0.025.

Sensitivity analyses included repetition of the primary outcome analysis using the ITT population with different missing data models. These included a "best case" (missing set to non-HED), "worst" case (missing set to HED), "conservative case" (missing in control arm set to non-HED, missing in intervention arm set to HED) and multiple imputation with 50 imputed data sets.

To explore differential intervention effects on the primary measures, pre-specified interaction terms were fitted between trial arm and baseline measures thought to predict the effect of intervention on primary outcomes. These were: age (months) at baseline; gender; socioeconomic status (proportion of students in receipt of FSM tertile split); alcohol use behaviour at baseline – age of initiation, use of alcohol in the year prior to baseline, context of use (abstainer/supervised/ unsupervised); and in NI, Grammar/Secondary school.

Process outcomes were assessed across eight pre-specified domains (including intervention acceptability and assessment of the content of EAN), using nine data sources. Methodologies included focus groups with students, an online survey with teachers, and interviews with senior school staff and stakeholders. Fidelity and completeness of delivery were assessed using bespoke tools and calculation of participation rates at the parent/carer evening.

For peer review only - http://bmjopen.bmj.com/site/about/guidelines.xhtml

Data cleaning, data management and preliminary analysis were undertaken using IBM SPSS version 20+. Mplus 7.11 was used for all analyses and Stata/IC 12.0 was used to verify Mplus models and generate odds ratios (OR).

The trial was registered, number ISRCTN47028486.

Ethics approval and consent to participate

The research was approved by Liverpool John Moores University Research Ethics Committee (11/HEA/097). Participants were eligible students in the randomised schools, who consented to participate. Consent was obtained from school head-teachers/principals before randomisation. Consent was obtained from participants and their parents/guardians after randomisation. This was through an opt-out method as opt-in written consent was not required by the ethics committee.

Results

Fig 1 shows participant flow through the trial. School recruitment began in November 2011 and ended in January 2012. As this was a cRCT of an intervention taking place across several years, student numbers refer to those who completed the questionnaire at each data collection period. No participant or parent/carer requested data were retrospectively removed from analysis. Multiple data collection 'mop up' visits were undertaken with schools, and attrition represents students who were absent on data collection days rather than formal drop out. Of the full sample (those who completed a questionnaire at either baseline or 12 months, N=12,738), 10,405 also completed the questionnaire at 33 months (81.7%). There was a higher attrition rate amongst

For peer review only - http://bmjopen.bmj.com/site/about/guidelines.xhtml

students who were male (19.0%), in receipt of FSM (25.8%), and had used alcohol at baseline (25.4%). There was little difference in attrition between the control and intervention arms of the trial (around one percentage point difference). Attrition also varied by location, with a higher rate in Scotland (24.0%) compared to NI (15.0%). Across schools attrition varied from 1.5% to 32.0%. There were no unintended harms or adverse effects reported.

INSERT FIGURE 1 HERE

Baseline data collection took place in June 2012 with the following follow up data collection points: 12 months (after delivery of phase one of the classroom component); 24 months (after delivery of the parental intervention and phase two of the classroom component); and 33 months. The trial ended as planned after final data collection and analysis.

Baseline characteristics of students (n=11,316) are presented in Table 2. No significant differences in baseline characteristics were detected between control and intervention arms. Overall parental/carer participation was low. A total of 319 parent(s)/carer(s) attended the intervention evenings in NI (9% of those eligible) and 63 parents attended in Scotland (2.5%). With respect to the follow-up mailed intervention, 1074 returns were received from parent(s)/carer(s) in NI (a 31% return) and 440 in Scotland (18%).

INSERT TABLE 2 HERE

For peer review only - http://bmjopen.bmj.com/site/about/guidelines.xhtml

Table 2. Baseline characteristics of students according to study condition.

	Control n ($\%_{valid}$)	Intervention n ($\%_{valid}$)
Total (n=11,316)	5567 (49.2)	5749 (50.8)
Gender		
Male	2787 (51.1)	2834 (50.0)
Female	2670 (48.9)	2829 (50.0)
Missing	*110*	*86*
Free School Meals		
No	4289 (77.3)	4436 (77.5)
Yes	1258 (22.7)	1290 (22.5)
Missing	*20*	*23*
Location		
NI	3469 (62.3)	3554 (61.8)
Scotland	2098 (37.7)	2198 (38.2)
Missing	*0*	*0*
HED[a]		
No	5082 (92.2)	5261 (92.4)
Yes	432 (7.8)	431 (7.6)
Missing	*53*	*57*
Ethnicity		
White	4492 (95.3)	4495 (94.5)
Non-white	248 (4.5)	293 (5.5)
Missing	*827*	*961*

Note: The percentages are calculated on the basis of the complete cases only.

[a] Assessed at baseline as consuming > 5 drinks in one or more episodes in the last 30 days.

For peer review only

For peer review only - http://bmjopen.bmj.com/site/about/guidelines.xhtml

Table 3 shows the count and percentages of respondents reporting drinking above the primary outcome threshold (≥6/≥4.5 units) at 33 months, and the adjusted model results by study arm (OR; Incidence rate ratio, IRR). Around one in 5 participants reported at least one episode in the last 30 days. The prevalence of episodes was around nine percentage points higher in the control group (26%) than in the intervention group (17%). Taking the within (pupil) level variance (fixed at 3.29) and the between (school) level variance (0.454 for the full sample), estimated using a null two level model, the corresponding ICC for the full sample was 0.121. Supplementary Tables S1and S2 show the full random intercept models for the primary outcomes at 33 months.

Table 3 Primary outcomes at 33 months by study group

	Unadjusted results		Adjusted model results	
	Control	Intervention		
	N ($\%_{valid}$)	N ($\%_{valid}$)	OR/IRR	95% CI
HED (frequency)				
None	3773 (74.4)	4281 (83.0)	0·60	0·49-0·73
One or more occasion	1300 (25.6)	879 (17.0)		
Missing	*1286*	*1219*		
ARH (frequency)				
None	3126 (60.7)	3408 (65.1)	0·92	0·78-1.05
One or more occasion	2020 (39.3)	1826 (34.9)		
Missing	*1213*	*1145*		
Median (IQR)	0 (2)	0 (3)		

OR, odds ratio; IRR, incidence rate ratio; HED, Heavy episodic drinking; ARH, Alcohol related harms.

For peer review only - http://bmjopen.bmj.com/site/about/guidelines.xhtml

Fig 2 displays the count of respondents reporting ARH at 33 months by study group. Around two thirds of students (63%) reported no alcohol-related harms. The median number of harms was equivalent in each study arm (0), while the interquartile range was smaller in the intervention arm than in the control arm (IQR = 2 and 3 respectively).

INSERT FIGURE 2 HERE

At the school level, the parameter estimates were significant for the intervention arm (estimate = -0.516, SE=0.102; p < 0.001). Schools in the intervention arm had lower levels of HED (their intercepts) than those in the control arm (OR = 0.596, 95% CI 0.490 – 0.725). This represents a significant intervention effect. However, with respect to ARH, the intervention indicator was non-significant suggesting no difference between the intervention and control schools (estimate -0.101, SE = 0.083; p = 0.222; IRR = 0.916, 95% CI 0.780 – 1.052). Across three of the sensitivity analysis models (best case; worst case; and multiple imputed data models) the intervention arm coefficient remained significant and retained the same sign for HED (i.e. being a school in the intervention arm was associated with having a lower intercept), while ARH remained non-significant. The only exception was the conservative case model, where both primary outcomes were non-significant.

When the primary measures were assessed at +24 months, as secondary outcomes, the intervention arm was significant at a 0.05 level (β=-0.241; p=0.041) in the HED model, but failed to reach the much stricter threshold used within this study

For peer review only - http://bmjopen.bmj.com/site/about/guidelines.xhtml

(p<0.025) (Supplementary Table S3). The intervention arm was also non-significant when the ARH outcome was assessed at +24 months (β=-0.144; p=0.22) (Supplementary Table S3). In all the other secondary outcomes, including those assessed at +33 months (Supplementary Table S4) and at +24 months (Supplementary Table S5), the intervention arm was non-significant.

Discussion

In a large cRCT we found that the STAMPP intervention reduced self-reported heavy episodic drinking (HED) in the past 30 days at 33 months follow-up from baseline, compared with education as normal (EAN), but not alcohol-related harms (ARH) associated with own drinking. There were no clear or consistent effects identified in planned secondary or sub-group analyses (age, gender, SES, alcohol use at baseline, location [Scotland vs NI]). It is possible that longer-term follow-up and/or emphasis on those drinking might reveal such effects, especially with regard to self-reported ARH, which were low in both control and intervention students. The intervention was well received by both pupils and teachers.

Key strengths of the trial were the large sample size (schools and students), low rates of attrition (no schools dropped out), and relatively high rates of matched data (>80%) across survey waves. This means that the analyses were sufficiently powered. There also appeared to be no comparator bias, as monitoring of delivery of EAN in intervention schools showed that this did not include alcohol education. A major limitation of the work was the failure to attract parents/carers to the brief intervention evening, despite the support of many of the schools. Although all intervention

For peer review only - http://bmjopen.bmj.com/site/about/guidelines.xhtml

students received a mailed follow up leaflet that reinforced the main messages of the parental intervention, relatively low rates of return of the parental questionnaire suggest that only a minority may have read the mailed information. In contrast, parental participation in the structurally similar (i.e. classroom and parental components) Swedish Örebro Prevention Program, and the Dutch Prevention of Alcohol use in Students (PAS) alcohol prevention programmes were relatively high.(24, 38) Because we chose a parental intervention based on one with face-to-face contact (21), we attempted to engage parents at school-based meetings. However, it is possible that the use of a DVD or the creation of a Web-based presentation could have served this purpose equally well.(22) Universal interventions such as STAMPP require a range of recruitment strategies as there will be different barriers to, and facilitators of, attendance in parental/carer-based actions. Research is therefore needed to assess the relative efficacy of recruitment strategies such as incentives, mass media campaigns, the removal of barriers to attendance (e.g. providing transport and childcare), and the use of key community recruiters (influential individuals and organisations).(39) Furthermore, it is also important to understand if some parent/carer subgroups (e.g. differentiated on child drinking risk) are more likely to respond to particular recruitment strategies, and if this will lead to recruitment biases.

Although we conducted an ITT analysis which helped to preserve sample size, the achieved participation rates are likely to reflect parental/carer attendance in routine UK practice.(40-42) This meant that we were unable to draw any confident inferences about the combined impact of the school and parental intervention (*cf (29)*), or the relative contribution of each component. In practical terms, this means that although the analysis presumed delivery of the combined intervention, discussions with

For peer review only - http://bmjopen.bmj.com/site/about/guidelines.xhtml

stakeholders about research findings and future delivery are likely to focus on the classroom component (i.e. culturally adapted SHAHRP). However, it is noteworthy that in the PAS programme (21), the classroom component alone did not produce changes in alcohol use behaviours, and these were only observed in pupils receiving the combined intervention. Subsequent mediation analysis of trial data suggested that reduced rate of frequency of drinking or weekly drinking, was mediated by changes in parental rules and attitudes towards alcohol (i.e. more strict rules and attitudes were developed). It is therefore important that similar analyses are undertaken to better understand mediators of behaviour change in STAMPP recipients. Other weaknesses of the study included the lack of blinding in intervention delivery and in some data collectors. It is plausible that lack of blinding in delivery may led to either under- or over-reporting of alcohol use due to social desirability biases, but using an EAN comparator meant that it was not possible to conceal intervention allocation from teachers, who received specialised training and curriculum materials, or pupils, who would typically receive little or no alcohol education in their usual school year. Lack of blinding in some data collectors may have also led to either under- or over-reporting of alcohol use due to social desirability biases, although the use of standardised data collection scripts mitigated against this.

Our primary outcome assessment relied on self-report, which may have led to inaccurate reporting of alcohol use through memory, social desirability, and other biases.(43) Although adolescent self-reported alcohol questionnaires are generally reliable,(44) there may be differences in reliability between early and late adolescence,(20) and studies of recanting in substance use surveys suggest that this may be an understudied bias in prevention research.(37) However, all students

For peer review only - http://bmjopen.bmj.com/site/about/guidelines.xhtml

received the same questionnaire and pictorial prompts, and the recall period for the primary outcome used in this study was the previous 30 days, and so if bias had existed, this would have been minimal, and equivalent across trial arms.

Although the classroom component of STAMPP was based on the SHAHRP programme, we did not detect a decrease in ARH. Previous studies of SHAHRP in Australia and NI using quasi-experimental designs found that decreases in self-reported ARH at 32 months were associated with intervention exposure.(23, 30) Differences with the findings of this trial may be related to factors such as methodology, pupil age, changes in the wider drinking culture and public health environment, or other unmeasured cohort effects. Whilst there is a relationship between HED in adolescence and health harms(1) we have planned further exploratory analyses which will investigate ARH, patterns of reporting, and sub group effects in more detail.

Although we are mindful of differences in school autonomy, governance and oversight, and acknowledge regional variability in alcohol use behaviours (e.g.(5)), we believe that the findings of this trial are likely to be applicable to other geographies. Schools enrolled in the trial were drawn from urban and more rural areas, and from across the socioeconomic gradient. Furthermore, sub group analyses showed that there were no differential intervention effects on the basis of school geography (i.e. NI vs Scotland).

Conclusions

For peer review only - http://bmjopen.bmj.com/site/about/guidelines.xhtml

The results of this large cRCT provide support for the effectiveness of a combined classroom and brief parental intervention for reducing HED, but not ARH, in young adolescents. Effects on ARH may manifest later, but further research would be required to clarify this.

Acknowledgements

As well as acknowledging the role played by participating schools and school children, the authors would like to acknowledge the support of the following people in this project: Séamus Mullin, Gerry Bleakney, Owen O'Neill (PHANI); Malachy Crudden (CCMS), Maura Kearney, and Fergal Doherty (Psychological Services, Glasgow); Kate Watson (Psychological Services, Inverclyde) and John Butcher and Sandy Cunningham (Education Services, Glasgow). The views and opinions expressed therein are those of the authors and do not necessarily reflect those of the NIHR-PHR, NIHR, NHS or the Department of Health.

For peer review only

A. **Author Contributions**: Sumnall had full access to all of the data in the study and takes responsibility for the integrity of the data and the accuracy of the data analysis. McKay wrote the first draft of the manuscript and subsequent versions, and submitted the final version; Sumnall was project PI, contributed to the first draft and subsequent iterations of the manuscript, and prepared the final version of the manuscript; Percy conducted the statistical analysis and contributed to manuscript drafts; Agus, Foxcroft, Cole, Murphy, Doherty, Harvey all contributed to drafts and approved the submission.

B. **Declaration of interests:** No personal competing interests declared. The sponsor University (LJMU) received and administered a payment from the alcohol

For peer review only - http://bmjopen.bmj.com/site/about/guidelines.xhtml

industry for printing of student workbooks in the Glasgow trial site only. Percy reported that he has previously received funding from the European Foundation of Alcohol Research (ERAB) in relation to the development of statistical models for longitudinal data (2008-2010). Foxcroft reported that his Department has previously received funding from the alcohol industry for unrelated prevention programme training work. Sumnall reported that his Department has previously received funding from the alcohol industry (indirectly via the industry funded Drinkaware charity) for unrelated primary research.

C. **Funding:** This trial was funded by the National Institute of Health Research (NIHR) Public Health Research (PHR) programme (project number 10/3002/09). The Public Health Agency of NI and Education Boards of Glasgow/Inverclyde provided some intervention costs. Diageo provided funds to print classroom workbooks for use only in the Glasgow Local Authority area. Remaining intervention costs were internally funded. The research and intervention funders had no involvement in intervention design; design and conduct of the study; collection, management, analysis, and interpretation of the data; and preparation, review or approval of the manuscript.

D. **Data:** Availability of data and materials: The datasets generated during and/or analysed during the current study are not yet publicly available due to the authors undertaking additional analyses and follow-on studies, but are available from the corresponding author on reasonable request.

For peer review only - http://bmjopen.bmj.com/site/about/guidelines.xhtml

Fig 1. School and participant flow diagram - STAMPP Trial. Analysis was conducted at 33 months on students who had completed each of the primary outcome measures. N = number of schools; n = student numbers

Fig 2. Count of school children reporting one or more alcohol related harms by study arm

For peer review only

References

1. Oesterle S, Hill KG, Hawkins JD, Guo J, Catalano RF, Abbott RD. Adolescent heavy episodic drinking trajectories and health in young adulthood. J Stud Alcohol. 2004;65(2):204-12.

2. Bellis MA, Morleo M, Hughes K, Downing J, Wood S, Smallthwaite L, et al. A cross-sectional survey of compliance with national guidance for alcohol consumption by children: measuring risk factors, protective factors and social norms for excessive and unsupervised drinking. BMC Public Health. 2010;10:8.

3. McMorris BJ, Catalano RF, Kim MJ, Toumbourou JW, Hemphill SA. Influence of family factors and supervised alcohol use on adolescent alcohol use and harms: similarities between youth in different alcohol policy contexts. J Stud Alcohol Drugs. 2011;72(3):418-28.

4. Livingston JA, Testa M, Hoffman JH, Windle M. Can parents prevent heavy episodic drinking by allowing teens to drink at home? Addict Behav. 2010;35(12):1105-12.

For peer review only - http://bmjopen.bmj.com/site/about/guidelines.xhtml

5. Maimaris W, McCambridge J. Age of first drinking and adult alcohol problems: systematic review of prospective cohort studies. J Epidemiol Community Health. 2014;68(3):268-74.

6. Jones L, McCoy E, Bates G, Bellis MA, Sumnall HR. Understanding the Alcohol Harm Paradox. London: Alcohol Research UK; 2015.

7. Jones L, Bates G, McCoy E, Bellis MA. Relationship between alcohol-attributable disease and socioeconomic status, and the role of alcohol consumption in this relationship: a systematic review and meta-analysis. BMC Public Health. 2015;5.

8. Marmot M. Social determinants of health inequalities. Lancet. 2005;365(9464):1099-104.

9. Foxcroft DR, Tsertsvadze A. Universal family-based prevention programs for alcohol misuse in young people. Cochrane Database Syst Rev. 2011(9):CD009308.

10. Foxcroft DR, Tsertsvadze A. Universal multi-component prevention programs for alcohol misuse in young people. Cochrane Database Syst Rev. 2011(9):CD009307.

11. Foxcroft DR, Tsertsvadze A. Universal school-based prevention programs for alcohol misuse in young people. Cochrane Database Syst Rev. 2011(5):CD009113.

12. Nation M, Crusto C, Wandersman A, Kumpfer KL, Seybolt D, Morrissey-Kane E, et al. What works in prevention. Principles of effective prevention programs. The American psychologist. 2003;58(6-7):449-56.

13. Faggiano F, Vigna-Taglianti FD, Versino E, Zambon A, Borraccino A, Lemma P. School-based prevention for illicit drugs use: a systematic review. Prev Med. 2008;46(5):385-96.

For peer review only - http://bmjopen.bmj.com/site/about/guidelines.xhtml

14. Spoth R, Greenberg M, Turrisi R. Preventive interventions addressing underage drinking: state of the evidence and steps toward public health impact. Pediatrics. 2008;121 Suppl 4:S311-36.

15. Flynn AB, Falco M, Hocini S. Independent evaluation of middle school–based drug prevention curricula: A systematic review. JAMA Pediatrics. 2015;169(11):1046-52.

16. National Institute for Health and Care Excellence. Alcohol: school-based interventions London; 2007.

17. Ryan SM, Jorm AF, Lubman DI. Parenting factors associated with reduced adolescent alcohol use: a systematic review of longitudinal studies. Aust N Z J Psychiatry. 2010;44(9):774-83.

18. Vakalahi HF. Adolescent substance use and family-based risk and protective factors: a literature review. Journal of drug education. 2001;31(1):29-46.

19. Yap, MBH., Cheong, TWK., Zaravinos,-Tsakos, F, Lubman, DI, Jorm, AF. Modifiable parenting factors associated with adolescent alcohol misuse: a systematic review and meta-analysis of longitudinal studies. Addiction. 2017;112:1142-1162.

20. Koning IM, Engels RC, Verdurmen JE, Vollebergh WA. Alcohol-specific socialization practices and alcohol use in Dutch early adolescents. J Adolesc. 2010;33(1):93-100.

21. Koning IM, Vollebergh WA, Smit F, Verdurmen JE, Van Den Eijnden RJ, Ter Bogt TF, et al. Preventing heavy alcohol use in adolescents (PAS): cluster randomized trial of a parent and student intervention offered separately and simultaneously. Addiction. 2009;104(10):1669-78.

22. Newton, NC, Champion, KE, Slade, T, Chapman, C, Stapinski, L, Koning, I, Tonks, Z, Teesson, M. A systematic review of combined student- and parent-based

For peer review only - http://bmjopen.bmj.com/site/about/guidelines.xhtml

programs to prevent alcohol and other drug use among adolescents. Drug. Alcohol Rev. 2017;36(3):337-351.

23. McBride N, Farringdon F, Midford R, Meuleners L, Phillips M. Harm minimization in school drug education: final results of the School Health and Alcohol Harm Reduction Project (SHAHRP). Addiction. 2004;99:278-91.

24. Koutakis N, Stattin H, Kerr M. Reducing youth alcohol drinking through a parent-targeted intervention: the Orebro Prevention Program. Addiction. 2008;103(10):1629-37.

25. McKay MT, Sumnall HR, Percy A, Cole JC. Self-Esteem and Self-Efficacy: associations with alcohol consumption in a sample of Adolescents in Northern Ireland. Drugs: Education, Prevention, and Policy. 2012;19(1):72-80.

26. McKay MT, Cole JC, Sumnall HR. Teenage Thinking on Teenage Drinking: 15- to 16- year olds' experiences of alcohol in Northern Ireland
Drugs: Education, Prevention, and Policy. 2011;18(5):323-32.

27. McKay MT, Sumnall HR, Goudie AJ, Percy A, Field M, Cole JC. What differentiates Adolescent Problematic Drinkers from their Peers? Results from a cross sectional study in Northern Irish School Children. Drugs: Education, Prevention, and Policy. 2011;18(3):187-99.

28. Farringdon F, McBride N, Midford R. School Health and Alcohol Harm Reduction Project: Formative development of intervention materials and processes. International Journal of Health Promotion and Education. 1999;37(4):137-43.

29. Koning IM, van den Eijnden RJ, Engels RC, Verdurmen JE, Vollebergh WA. Why target early adolescents and parents in alcohol prevention? The mediating effects of self-control, rules and attitudes about alcohol use. Addiction. 2011;106(3):538-46.

For peer review only - http://bmjopen.bmj.com/site/about/guidelines.xhtml

30. McKay MT, McBride NT, Sumnall HR, Cole JC. Reducing the harm from adolescent alcohol consumption: results from an adapted version of SHAHRP in Northern Ireland. Journal of Substance Use. 2012;17(2):98-121.

31. Donaldson L. Guidance on the consumption of alcohol by children and young people. London: Department of Health; 2009.

32. Council For the Curriculum, Examinations, and Assessment (CCEA) (2015) Drugs Guidance for Schools in Northern Ireland. Belfast: CCEA http://ccea.org.uk/sites/default/files/docs/curriculum/area_of_learning/pdmu/drugs/Drugs_Guidance_for_Schools.pdf

33. Scottish Government (2008). Curriculum For Excellence. Building the Curriculum 3: A Framework for Learning and Teaching. Edinburgh: Scottish Government.

34. van der Vorst H, Engels RC, Meeus W, Dekovic M. The impact of alcohol-specific rules, parental norms about early drinking and parental alcohol use on adolescents' drinking behavior. J Child Psychol Psychiatry. 2006;47(12):1299-306.

35. Koning IM, Van den Eijnden RJ, Glatz T, Vollebergh WA. Don't Worry! Parental Worries, Alcohol-Specific Parenting and Adolescents' Drinking. Cognitive Therapy Research. 2013;37(1079-1088).

36. McBride N, Midford R, Farringdon F, Phillips M. Early results from a school alcohol harm minimization study: the School Health and Alcohol Harm Reduction Project. Addiction. 2000;95:1021-42.

37. Percy A, McAlister S, Higgins K, McCrystal P, Thornton M. Response consistency in young adolescents' drug use self-reports: A recanting rate analysis. Addiction. 2005;100(2):189-96.

For peer review only - http://bmjopen.bmj.com/site/about/guidelines.xhtml

38. Bodin MC, Strandberg AK. The Orebro prevention programme revisited: a cluster-randomized effectiveness trial of programme effects on youth drinking. Addiction. 2011;106(12):2134-43.

39. Segrott J. Recruitment and group composition strategies for family-based substance misuse prevention interventions: an exploratory evaluation. Journal of Children's Services. 2013;8(2):89-109.

40. Caria MP, Faggiano F, Bellocco R, Galanti MR, Group EU-DS. Classroom characteristics and implementation of a substance use prevention curriculum in European countries. Eur J Public Health. 2013;23(6):1088-93.

41. Prinz RJ, Smith EP, Dumas JE, Laughlin JE, White DW, Barron R. Recruitment and retention of participants in prevention trials involving family-based interventions. Am J Prev Med. 2001;20(1 Suppl):31-7.

42. Bauman KE, Ennett ST, Foshee VA, Pemberton M, Hicks K. Correlates of participation in a family-directed tobacco and alcohol prevention program for adolescents. Health Educ Behav. 2001;28(4):440-61.

43. Leigh BC, Gillmore MR, Morrison DM. Comparison of diary and retrospective measures for recording alcohol consumption and sexual activity. J Clin Epidemiol. 1998;51(2):119-27.

44. Lintonen T, Ahlstrom S, Metso L. The reliability of self-reported drinking in adolescence. Alcohol and alcoholism (Oxford, Oxfordshire). 2004;39(4):362-8.

List of supplementary information table captions

Table S1. Primary outcome (HED) outcome analysis at +33 months

Table S2. Primary outcome (ARH) outcome analysis at + 33 months

For peer review only - http://bmjopen.bmj.com/site/about/guidelines.xhtml

Table S3. Secondary analysis: primary outcomes at +24 month

Table S4. Secondary outcomes at +33 months

Table S5. Secondary outcomes at +24 months

For peer review only

For peer review only - http://bmjopen.bmj.com/site/about/guidelines.xhtml

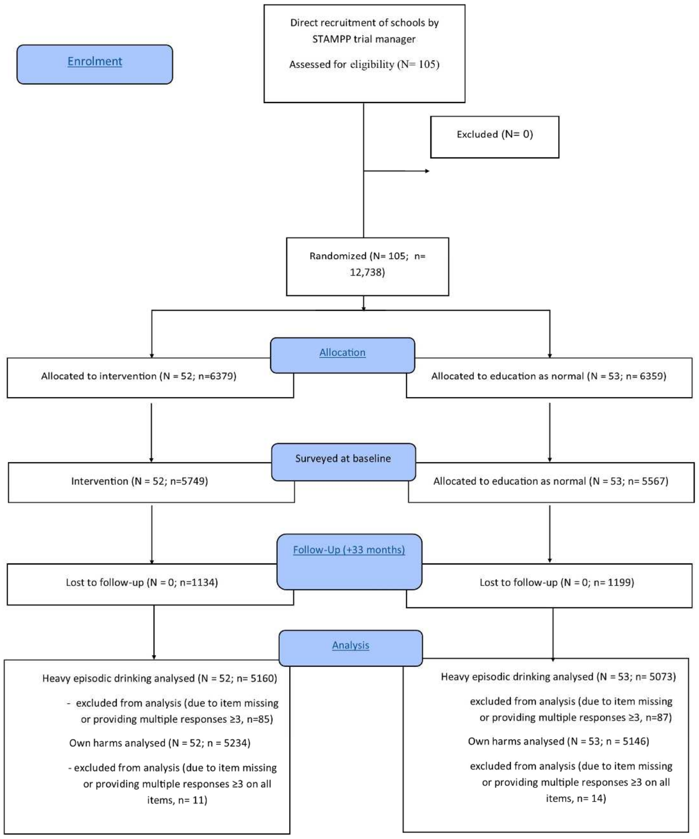

School and participant flow diagram - STAMPP Trial. Analysis was conducted at 33 months on students who had completed each of the primary outcome measures. N = number of schools; n = student numbers

209x297mm (300 x 300 DPI)

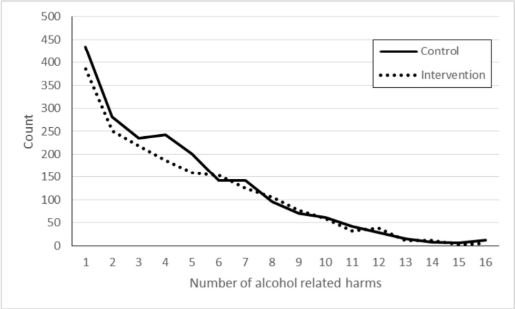

Count of school children reporting one or more alcohol related harms by study arm

209x297mm (300 x 300 DPI)

ONLINE SUPPLEMENTARY MATERIAL

INTERVENTION CONTENT

The Classroom component of the intervention was composed of six lessons in the second year of High school (Phase one), and four lessons in the third year (Phase two). The content of these is detailed below.

Phase 1

Lesson 1: Alcohol True or False (10 statements); Introduction to what is meant by ‘Units’ of alcohol; Introduction to the extent of harm that alcohol misuse can cause.

Lesson 2: Making Choices – why people choose to drink (and assessing the merit of those choices); Making Choices – why people may choose not to drink; Introduction to Alcohol and the Body.

Lesson 3: Units of Alcohol – more detail including unit content of drinks; Relating consumption to consequences; Short Quiz to recap information.

Lesson 4: Blood Alcohol Concentration; Alcohol harms in various societal contexts (with other drugs, in families, in communities, driving, and sexual behaviour).

Lesson 5: Exercise – ‘What would you do to reduce harms?’ Critical examination of alcohol and the Media.

Lesson 6: Real Life Scenarios, plus recap.

Phase 2

Lesson 1: Brief recap from previous year; Alcohol and the Body – long term versus short term; Quiz.

Lesson 2: A night out – examining dangers, laws, problems, pressures and consequences.

Lesson 3: Vulnerability – two scenarios examined from the point of view of ‘victim’, friends, and ‘perpetrator’; Planning for a safe night out with friends.

Lesson 4: Ranking Risk; What would you advise a friend to do?

For peer review only - http://bmjopen.bmj.com/site/about/guidelines.xhtml

STAMPP – FULL PRIMARY OUTCOME MODELS, SECONDARY OUTCOMES AND SUBGROUP ANALYSES

FULL PRIMARY OUTCOME MODELS

For reasons of space, the full primary outcome models were not presented in the main text. Table S1 presents the parameter estimates from a two level random intercepts logistic regression model for the heavy episodic drinking (HED) primary outcome at T3.

Table S1. Primary outcome (HED) outcome analysis at + 33 months

	Estimate	S.E.	OR	P value
**ITT Complete case analysis**				
Within level				
Baseline HED	1.395	0.093	4.036	<0.001
Between Level				
Intervention Arm	-0.516	0.102		<0.001
Free School Meals (tertile)	0.239	0.073		0.001
School Type				
Boys School Dummy	-0.186	0.200		0.35
Girls School Dummy	-0.546	0.266		0.04
Location (NI)	0.422	0.109		<0.001
School level residual variance	0.176	0.035		<0.001
Threshold (BngT3$1)	1.574	0.124		<0.001

Table S2 gives the parameter estimates from a two level random intercepts negative binomial model for the drinking harms primary outcome at T3.

Table S2. Primary outcome (ARH) outcome analysis at + 33 months

	Estimate	S.E.	P value
**Complete case analysis**			
Within level			
Baseline Harms	0.211	0.011	<0.001
Between Level			
Intervention Arm	-0.101	0.083	0.222
Free School Meals (tertile)	0.168	0.061	0.006
School Type			
Boys School Dummy	-0.083	0.204	0.685
Girls School Dummy	-0.380	0.236	0.107
Location	0.433	0.082	<0.001
Residual variances	0.115	0.026	<0.001
Intercept (HarmsT3)	-0.042	0.093	0.649
Dispersion (HarmsT3)	3.563	0.207	<0.001

SECONDARY OUTCOMES

For peer review only - http://bmjopen.bmj.com/site/about/guidelines.xhtml

A range of secondary outcomes were also examined within the study. These included the primary outcomes assessed at T2:

Heavy episodic drinking (HED) (T2): Self-reported alcohol use defined as self-reported consumption of >5 drinks, assessed at +24 months (T2) from baseline. This was dichotomised at none/one or more occasions. This outcome was assessed via a two level random intercepts logistic regression model. Around 12.4% of respondents reported HED at T2 using this measure. In the intervention arm HED was reported by 10.9% (N=573) and in the control arm by 13.9% (N=722).

Alcohol related harms (T2): The number of self-reported harms (harms caused by own drinking) assessed at +24 months (T2) from baseline. Items included harms such as getting into a physical fight or being sick after drinking. The outcome was a count of the number of discrete harms reported (0-16) and was assessed by a two level random intercepts negative binomial model. In the intervention arm 74.3% reported no drinking harms, while in the control arm 71.5% reported no harms.

In addition, a number of secondary outcomes at T3 and T2 were also examined, including:

Lifetime drinking (T3): Whether the pupils had ever consumed a full drink of alcohol at +33 months (T3) (two level random intercepts logistic regression model).

Last year drinking (T3): Whether the pupils had consumed a full drink of alcohol in the last year, assessed at +33 months (T3) (two level random intercepts logistic regression model).

For peer review only - http://bmjopen.bmj.com/site/about/guidelines.xhtml

Last month Drinking (T3): Whether the pupils had consumed a full drink of alcohol in the last month, assessed at +33 months (T3) (two level random intercepts logistic regression model).

Harm from others (T3 and T2): The number of self-reported harms experienced that were the result of other people's drinking, assessed at both +33 months (T3) and +24 months (T2) from baseline (two level random intercepts negative binomial models). Harms included being hit or having property damaged by someone who had been drinking.

Age of onset (T3 and T2): Self-reported age at which respondent first consumed a full drink, assessed at both +33 months (T3) and +24 months (T2) from baseline (two level random intercepts Cox regression model).

Unsupervised drinking (T3 and T2): Whether the pupils were permitted, by their parents(s), to consume alcohol (with small group of friends or at parties) with no adult present, assessed at both +33 months (T3) and +24 months (T2) from baseline (two level random intercepts logistic regression model).

Number of drinks consumed (T3 and T2): Pupils were asked whether they usually drank from a range of different alcohol drinks (beer, alcopops, spirits cider, wine, *Buckfast* [a popular brand of fortified wine, with caffeine], others) and if so, how much did they usually drink. The values for each drink were summed together to give a total. As the underlying items continued decimals the total value was multiplied by 10 to create whole numbers.

The secondary outcome analysis also included covariates at level 1 (individual) and level 2 (school) where appropriate:

For peer review only - http://bmjopen.bmj.com/site/about/guidelines.xhtml

The models use for the secondary outcome were similar to those employed in the primary outcome analysis with a single level one covariate, and the treatment indicator and stratification variables used in the randomisation as level two covariates.

Level 1 covariate

Relevant baseline drinking variable (T0): For each outcome, the corresponding baseline observations were included in the model. Mean imputation was used to impute values for those respondents who were missing on this variable. The only model not to include a baseline covariate was age of onset.

Level 2 covariates

Treatment Arm: This was a binary covariate in which schools in the control arm were coded 0 and schools in the intervention arm were coded 1.

Free school meals (Randomisation stratification factor): Schools were classified into three groups based on free school meal provision. The allocation was based on a tertile split based on information provided by head teachers on the proportion of pupils in receipt of free school meals: *Low* Free School Meal Provision (0-15.4%), *Moderate* Free School Meal Provision (15.5-30.4%), *High* Free School Meal Provision (30.5% and above).

School type (Randomisation stratification factor): Given the larger number of schools in Northern Ireland, an additional stratification factor was used in the randomisation. This was school type (all boys' school/ all girls' school/coeducation school). Schools in

For peer review only - http://bmjopen.bmj.com/site/about/guidelines.xhtml

Glasgow/Inverclyde were all assigned to the co-education type. This indicator was used represented by two dummy variables (co-education was the comparison category).

Location: A dummy variable was generated to indicate the location of the schools (Northern Ireland/Scotland).

Results from the analysis of secondary outcomes

Table S3 presents the random intercept models for the primary outcomes at +24 months. The baseline measures were significant, as was location. For the HED outcomes both free school meals (tertile split) and school type were significant. The intervention arm was significant at a 0.05 level (β=-0.241; p=0.041). However, it failed to reach the much stricter threshold used in the primary analysis (0.025). It should be noted that the HED indicator used at +33 months, and as specified in the DAP, was different that that used at +24 months. In particular, this measure did not use gender specific splits, referred to drinks rather than units, and did not provide any visual guides to help with the estimation of amount consumed. This suggests that the significant intervention effect may have been partly dependent on the precision of the measurement instrument used to collect the primary outcome data. The age at which differences in HED were assessed may have been important when assessing intervention outcomes.

Table S3. Secondary analysis: primary outcomes at +24 months

	Estimate	S.E.	OR	P value
**HED T2 (ITT CC population, logistic model)**				
Within level				
Baseline HED	1.891	0.101	6.623	<0.001
Between Level				
Treatment Arm	-0.241	0.118		0.041
Free School Meals (tertile)	0.308	0.079		<0.001

For peer review only - http://bmjopen.bmj.com/site/about/guidelines.xhtml

School Type			
Boys School Dummy	-0.708	0.297	0.02
Girls School Dummy	-0.608	0.186	0.001
Location	0.732	0.134	<0.001
Residual variance	0.214	0.047	<0.001
Threshold (BngT2$1)	2.698	0.144	<0.001
**Harms to Self T2 (ITT CC population, negative binomial model)**			
Within level			
Baseline Harms drinking	0.297	0.016	<0.001
Between Level			
Treatment Arm	-0.144	0.118	0.22
Free School Meals (tertile)	0.162	0.086	0.06
School Type			
Boys School Dummy	-0.247	0.302	0.42
Girls School Dummy	-0.246	0.200	0.22
Location	0.716	0.132	<0.001
Residual variance	0.267	0.054	<0.001
Intercepts (SHarmsT2)	-0.779	0.133	<0.001
Dispersion	4.478	0.304	<0.001

Table S4 presents the outcome models for the additional secondary outcomes assessed at T3.

The treatment indicator was not significant in any of these models.

Table S4. Secondary outcomes at +33 months

	Estimate	S.E.	OR	P value
**Lifetime drinking T3 (ITT CC population, logistic model)**				
Within level				
Baseline HED	2·070	0·081	7·922	<0·001
Between Level				
Treatment Arm	-0·125	0·102		0·22
Free School Meals (tertile)	0·040	0·070		0·57
School Type				
Boys School Dummy	-0·182	0·209		0·384
Girls School Dummy	-0·501	0·233		0·031
Location	0·597	0·113		<0·001
Residual variance	0·209	0·035		<0·001
Threshold (LifeT3$1)	0·419	0·114		<0·001

Table S4. Secondary outcomes at +33 months (cont.)

	Estimate	S.E.	OR	P value
**Last year drinking T3 (ITT CC population, logistic model)**				
Within level				
Baseline Last year drinking	1·822	0·086	6·187	<0·001
Between Level				
Treatment Arm	-0·126	0·096		0·19
Free School Meals (tertile)	0·011	0·065		0·87

For peer review only - http://bmjopen.bmj.com/site/about/guidelines.xhtml

School Type				
Boys School Dummy	-0·176	0·211		0·40
Girls School Dummy	-0·401	0·229		0·08
Location	0·615	0·105		<0·001
Residual variances	0·177	0·032		<0·001
Threshold (LYearT3$1)	0·485	0·103		<0·001
**Last month drinking T3 (ITT CC population, logistic model)**				
Within level				
Baseline Last month drinking	1·329	0·114	3·779	<0·001
Between Level				
Treatment Arm	-0·149	0·094		0·11
Free School Meals (tertile)	0·114	0·069		0·10
School Type				
Boys School Dummy	-0·333	0·213		0·12
Girls School Dummy	-0·330	0·237		0·16
Location	0·381	0·104		<0·001
Residual variances	0·148	0·028		<0·001
Threshold (LMonthT3$1)	1·459	0·102		<0·001
**Harms from others drinking T3 (ITT CC population, Neg Bin model)**				
Within level				
Baseline Harms (others)	0·330	0·016		<0·001
Between Level				
Treatment Arm	0·000	0·057		0·10
Free School Meals (tertile)	0·077	0·042		0·07
School Type				
Boys School Dummy	0·117	0·116		0·31
Girls School Dummy	-0·070	0·172		0·68
Location	0·167	0·063		0·01
Residual variance	0·050	0·014		<0·001
Dispersion	1·301	0·071		<0·001
Intercept	-0·733	0·061		<0·001
**Age of onset T3 (ITT CC population, Cox regression model)**				
Between Level				
Treatment Arm	-0·095	0·067		0·16
Free School Meals (tertile)	0·054	0·047		0·25
School Type				
Boys School Dummy	-0·299	0·146		0·04
Girls School Dummy	-0·407	0·145		0·01
Location	0·344	0·075		<0·001
Residual variance	0·097	0·017		< 0·001

Table S4. Secondary outcomes at +33 months (cont.)

	Estimate	S.E.	OR	P value
**Unsupervised drinking T3 (ITT CC population Logistic model)**				
Within level				
Baseline unsupervised drinking	1.782	0.091	5.940	<0.001
Between Level				
Treatment Arm	-0.142	0.092		0.123
Free School Meals (tertile)	0.128	0.067		0.058

For peer review only

For peer review only - http://bmjopen.bmj.com/site/about/guidelines.xhtml

School Type				
Boys School Dummy	0.002	0.207		0.992
Girls School Dummy	-0.236	0.236		0.318
Location	0.564	0.102		<0.001
Residual variance	0.148	0.029		<0.001
Threshold (Unsuper$1)	0.148	0.029		<0.001
**Number of drinks T3 (ITT CC population NB model)**				
Within level				
Baseline number of drinks	0.126	0.009		<0.001
Between Level				
Treatment Arm	-0.078	0.075		0.297
Free School Meals (tertile)	0.123	0.048		0.011
School Type				
Boys School Dummy	-0.277	0.181		0.127
Girls School Dummy	-0.167	0.177		0.346
Location	0.363	0.075		<0.001
Residual variances	0.073	0.020		<0.001
Intercept (NumDrkT3)	3.521	0.082		<0.001
Dispersion (NumDrkT3)	5.371	0.306		<0.001

Note: The logistic regression multilevel models were estimated using a logit link function and the MLR estimator. The Cox regression model uses a non-parametric baseline hazard function and a profile likelihood estimation method

Table S5 presents the models for the secondary outcomes assessed at T2. Again, the treatment indicator was not significant in any of these models.

Table S5. Secondary outcomes at +24 months

	Estimate	S.E.	OR	P value
**Harms from others drinking T2 (ITT CC population, Neg Bin model)**				
Within level				
Baseline Harms (others)	0·421	0.017		<0.001
Between Level				
Treatment Arm	-0·058	0.060		0.33
Free School Meals (tertile)	0·132	0.044		0.003
School Type				
Boys School Dummy	0·144	0.108		0.18
Girls School Dummy	0·075	0.119		0.53
Location	0·255	0.071		<0.001
Residual variance	0·058	0.011		<0.001
Dispersion	1·032	0.078		<0.001
Intercept	-1·079	0.069		<0.001

Table S5. Secondary outcomes at +24 months

**Age of onset T2 (ITT CC population, Cox regression model)**			
Between Level			
Treatment Arm	-0·055	0.074	0.46
Free School Meals (tertile)	0·084	0.048	0.08
School Type			
Boys School Dummy	-0·528	0.197	0.007

For peer review only - http://bmjopen.bmj.com/site/about/guidelines.xhtml

For peer review only

Girls School Dummy	-0·453	0.169		0.007
Location	0·408	0.083		<0.001
Residual variance	0·176	0.028		<0.01
**Unsupervised drinking T2 (ITT CC population, Logistic model)**				
Within level				
Baseline unsupervised drinking	2·114	0.097	8.285	<0.001
Between Level				
Treatment Arm	-0·087	0.100		0.39
Free School Meals (tertile)	0·166	0.066		0.01
School Type				
Boys School Dummy	-0·306	0.217		0.16
Girls School Dummy	-0·207	0.135		0.12
Location	0·669	0.112		<0.001
Residual variance	0·170	0.038		<0.001
Threshold (Unsuper$1)	1·883	0.118		<0.001
**Number of drinks T2 (ITT CC population, NB model)**				
Within level				
Baseline unsupervised	0·170	0.013		<0.001
Between Level				
Treatment Arm	-0·088	0.096		0.36
Free School Meals (tertile)	0·125	0.068		0.07
School Type				
Boys School Dummy	-0·574	0.259		0.03
Girls School Dummy	-0·181	0.147		0.22
Location	0·583	0.105		<0.001
Residual variances	0·153	0.035		<0.001
Intercept (NumDrkT2)	2·836	0.106		<0.001
Dispersion (NumDrkT2)	5·671	0.340		<0.001

Note: The logistic regression multilevel models were estimated using a logit link function and the MLR estimator. The Cox regression model uses a non-parametric baseline hazard function and a profile likelihood estimation method

Subgroup analyses

To explore differential treatment effects on the primary and secondary outcome measures, pre-specified interaction terms were fitted between trial arm and baseline measures thought to predict the effect of treatment. These were:

- Age, in months, of pupil at baseline;
- Gender;
- Socioeconomic status (using the proportion of free school meals indicator);
- Alcohol use behaviour at baseline – ever use, last year use, age of onset, and context of use (abstainer/supervised/unsupervised);
- and in NI, a Grammar/Secondary school analysis.

For peer review only - http://bmjopen.bmj.com/site/about/guidelines.xhtml

Both the relevant covariate and interaction term were included in the model as a level 1 (within level) covariates. In all the subgroup analysis models estimated the corresponding interaction terms were all non-significant.

For peer review only

For peer review only - http://bmjopen.bmj.com/site/about/guidelines.xhtml

Table S1: CONSORT 2010 checklist of information to include when reporting a cluster randomised trial

Section/Topic	Item No	Standard Checklist item	Extension for cluster designs	Page No *
**Title and abstract**				
	1a	Identification as a randomised trial in the title	Identification as a cluster randomised trial in the title	1
	1b	Structured summary of trial design, methods, results, and conclusions (for specific guidance see CONSORT for abstracts)[1,2]	See table 2	2
**Introduction**				4
**Background and objectives**	2a	Scientific background and explanation of rationale	Rationale for using a cluster design	6
	2b	Specific objectives or hypotheses	Whether objectives pertain to the the cluster level, the individual participant level or both	5, 11
**Methods**				6
**Trial design**	3a	Description of trial design (such as parallel, factorial) including allocation ratio	Definition of cluster and description of how the design features apply to the clusters	6
	3b	Important changes to methods after trial commencement (such as eligibility criteria), with reasons		N/A
**Participants**	4a	Eligibility criteria for participants	Eligibility criteria for clusters	6-7
	4b	Settings and locations where the data were collected		6
**Interventions**	5	The interventions for each group with sufficient details to allow replication, including how and when they were actually administered	Whether interventions pertain to the cluster level, the individual participant level or both	8 & Table 1
**Outcomes**	6a	Completely defined pre-specified primary and secondary outcome measures, including how and	Whether outcome measures pertain to the cluster level, the individual participant level or both	11

		when they were assessed		
	6b	Any changes to trial outcomes after the trial commenced, with reasons		12
**Sample size**	7a	How sample size was determined	Method of calculation, number of clusters(s) (and whether equal or unequal cluster sizes are assumed), cluster size, a coefficient of intracluster correlation (ICC or *k*), and an indication of its uncertainty	12
	7b	When applicable, explanation of any interim analyses and stopping guidelines		**N/A**
**Randomisation:**				
**Sequence generation**	8a	Method used to generate the random allocation sequence		**7**
	8b	Type of randomisation; details of any restriction (such as blocking and block size)	Details of stratification or matching if used	7
**Allocation concealment mechanism**	9	Mechanism used to implement the random allocation sequence (such as sequentially numbered containers), describing any steps taken to conceal the sequence until interventions were assigned	Specification that allocation was based on clusters rather than individuals and whether allocation concealment (if any) was at the cluster level, the individual participant level or both	7
**Implementation**	10	Who generated the random allocation sequence, who enrolled participants, and who assigned participants to interventions	Replace by 10a, 10b and 10c	7
	10a		Who generated the random allocation sequence, who enrolled clusters, and who assigned clusters to interventions	6
	10b		Mechanism by which individual participants were included in clusters for the purposes of the trial (such as complete	7

For peer review only

			enumeration, random sampling)	
	10c		From whom consent was sought (representatives of the cluster, or individual cluster members, or both), and whether consent was sought before or after randomisation	7
**Blinding**	11a	If done, who was blinded after assignment to interventions (for example, participants, care providers, those assessing outcomes) and how		**7-8**
	11b	If relevant, description of the similarity of interventions		**11**
**Statistical methods**	12a	Statistical methods used to compare groups for primary and secondary outcomes	How clustering was taken into account	12
	12b	Methods for additional analyses, such as subgroup analyses and adjusted analyses		**14**
**Results**				15
**Participant flow (a diagram is strongly recommended)**	13a	For each group, the numbers of participants who were randomly assigned, received intended treatment, and were analysed for the primary outcome	For each group, the numbers of clusters that were randomly assigned, received intended treatment, and were analysed for the primary outcome	15 & Figure 1
	13b	For each group, losses and exclusions after randomisation, together with reasons	For each group, losses and exclusions for both clusters and individual cluster members	15 & Figure 1
**Recruitment**	14a	Dates defining the periods of recruitment and follow-up		**11**
	14b	Why the trial ended or was stopped		**16**
**Baseline data**	15	A table showing baseline demographic and clinical	Baseline characteristics for the individual and cluster levels as	15 & Table 2

For peer review

		characteristics for each group	applicable for each group	
**Numbers analysed**	16	For each group, number of participants (denominator) included in each analysis and whether the analysis was by original assigned groups	For each group, number of clusters included in each analysis	Table 2
**Outcomes and estimation**	17a	For each primary and secondary outcome, results for each group, and the estimated effect size and its precision (such as 95% confidence interval)	Results at the individual or cluster level as applicable and a coefficient of intracluster correlation (ICC or k) for each primary outcome	18
	17b	For binary outcomes, presentation of both absolute and relative effect sizes is recommended		**18-19**
**Ancillary analyses**	18	Results of any other analyses performed, including subgroup analyses and adjusted analyses, distinguishing pre-specified from exploratory		**20 & online supplementary material**
**Harms**	19	All important harms or unintended effects in each group (for specific guidance see CONSORT for harms[3])		**15**
**Discussion**				20
**Limitations**	20	Trial limitations, addressing sources of potential bias, imprecision, and, if relevant, multiplicity of analyses		**21-22**
**Generalisability**	21	Generalisability (external validity, applicability) of the trial findings	Generalisability to clusters and/or individual participants (as relevant)	23
**Interpretation**	22	Interpretation consistent with results, balancing benefits and harms, and considering other relevant evidence		**23-24**
**Other information**				
**Registration**	23	Registration number and		**14**

For peer review only

		name of trial registry	
**Protocol**	24	Where the full trial protocol can be accessed, if available	**6**
**Funding**	25	Sources of funding and other support (such as supply of drugs), role of funders	**24 and information included as part of journal submission process**

** Note: page numbers optional depending on journal requirements*

For peer review only

Table 2: Extension of CONSORT for abstracts1'2 to reports of cluster randomised trials

Item	Standard Checklist item	Extension for cluster trials
**Title**	Identification of study as randomised	**Identification of study as cluster randomised**
**Trial design**	Description of the trial design (e.g. parallel, cluster, non-inferiority)	
**Methods**		
**Participants**	Eligibility criteria for participants and the settings where the data were collected	**Eligibility criteria for clusters**
**Interventions**	Interventions intended for each group	
**Objective**	Specific objective or hypothesis	**Whether objective or hypothesis pertains to the cluster level, the individual participant level or both**
**Outcome**	Clearly defined primary outcome for this report	**Whether the primary outcome pertains to the cluster level, the individual participant level or both**
**Randomization**	How participants were allocated to interventions	**How clusters were allocated to interventions**
**Blinding (masking)**	Whether or not participants, care givers, and those assessing the outcomes were blinded to group assignment	
**Results**		
**Numbers randomized**	Number of participants randomized to each group	**Number of clusters randomized to each group**
**Recruitment**	Trial status[1]	
**Numbers analysed**	Number of participants analysed in each group	**Number of clusters analysed in each group**
**Outcome**	For the primary outcome, a result for each group and the estimated effect size and its precision	**Results at the cluster or individual participant level as applicable for each primary outcome**
**Harms**	Important adverse events or side effects	
**Conclusions**	General interpretation of the results	
**Trial registration**	Registration number and name of trial register	
**Funding**	Source of funding	

[1] Relevant to Conference Abstracts

REFERENCES

[1] Hopewell S, Clarke M, Moher D, Wager E, Middleton P, Altman DG, et al. CONSORT for reporting randomised trials in journal and conference abstracts. *Lancet* 2008, 371:281-283

[2] Hopewell S, Clarke M, Moher D, Wager E, Middleton P, Altman DG at al (2008) CONSORT for reporting randomized controlled trials in journal and conference abstracts: explanation and elaboration. *PLoS Med* 5(1): e20

[3] Ioannidis JP, Evans SJ, Gotzsche PC, O'Neill RT, Altman DG, Schulz K, Moher D. Better reporting of harms in randomized trials: an extension of the CONSORT statement. *Ann Intern Med* 2004; 141(10):781-788.

For peer review only
